# Systematic Review of the Literature and Evidence-Based Recommendations for Antibiotic Prophylaxis in Trauma: Results from an Italian Consensus of Experts

**DOI:** 10.1371/journal.pone.0113676

**Published:** 2014-11-20

**Authors:** Daniele Poole, Arturo Chieregato, Martin Langer, Bruno Viaggi, Emiliano Cingolani, Paolo Malacarne, Francesca Mengoli, Giuseppe Nardi, Ennio Nascimben, Luigi Riccioni, Ilaria Turriziani, Annalisa Volpi, Carlo Coniglio, Giovanni Gordini, on behalf of the Trauma Update Working Group

**Affiliations:** 1 U.O. Anestesia e Rianimazione, Ospedale S. Martino, Belluno, Italy; 2 SOD Anestesia e Area Intensiva CTO, AOU Careggi, Firenze, Italy; 3 Dipartimento di Anestesia e Rianimazione, Fondazione IRCCS Istituto Nazionale dei Tumori e Università degli Studi di Milano, Milano, Italy; 4 UOC Shock e Trauma, AO San Camillo-Forlanini, Roma, Italy; 5 U.O. Anestesia e Rianimazione - P.S., Azienda Ospedaliera Universitaria Pisana, Pisa, Italy; 6 U.O. Rianimazione 118, Ospedale Maggiore, Bologna, Italy; 7 Neurorianimazione Ospedale S. Maria di Ca' Foncello, Treviso, Italy; 8 Anestesia e Rianimazione 1, Azienda Ospedaliero-Universitaria di Parma, Parma, Italy; Purdue University, United States of America

## Abstract

**Background:**

Antibiotic prophylaxis is frequently administered in severe trauma. However, the risk of selecting resistant bacteria, a major issue especially in critical care environments, has not been sufficiently investigated. The aim of the present study was to provide guidelines for antibiotic prophylaxis for four different trauma-related clinical conditions, taking into account the risks of antibiotic-resistant bacteria selection, thus innovating previous guidelines in the field.

**Methods:**

The MEDLINE database was searched for studies comparing antibiotic prophylaxis to controls (placebo or no antibiotic administration) in four clinical traumatic conditions that were selected on the basis of the traumatic event frequency and/or infection severity. The selected studies focused on the prevention of early ventilator associated pneumonia (VAP) in comatose patients with traumatic brain injury, of meningitis in severe basilar skull fractures, of wound infections in long-bone open fractures. Since no placebo-controlled study was available for deep surgical site-infections prevention in abdominal trauma with enteric contamination, we compared 24-hour and 5-day antibiotic prophylaxis policies. A separate specific research focused on the question of antibiotic-resistant bacteria selection caused by antibiotic prophylaxis, an issue not adequately investigated by the selected studies. Randomised trials, reviews, meta-analyses, observational studies were included. Data extraction was carried out by one author according to a predefined protocol, using an electronic form. The strength of evidence was stratified and recommendations were given according to the Grading of Recommendations Assessment, Development and Evaluation (GRADE) criteria.

**Results:**

Uncertain evidence deserving further studies was found for two-dose antibiotic prophylaxis for early VAP prevention in comatose patients. In the other cases the risk of resistant-bacteria selection caused by antibiotic administration for 48 hours or more, outweighed potential benefits.

**Conclusions:**

When accounting for antibiotic-resistant bacteria selection we found no evidence in favour of antibiotic prophylaxis lasting two or more days in the studied clinical conditions.

## Introduction

In the attempt to investigate antibiotic prophylaxis in several trauma-related clinical conditions, taking into account the potential ecological risk, innovating previous approaches, in February 2013 a meeting of experts was held in Bologna in the context of the 8^th^ edition of the *Trauma Update and Organization* conference.

Infection is the consequence of contamination due to the contact between normally sterile tissues and microorganisms from the external environment or from internal sites. The inoculation occurs when the trauma breaks natural barriers against infections. All attempts to reduce the bacterial charge in the tissues should be made. Cleaning, irrigation, disinfection of wounds, followed by the appropriate early surgical treatment when indicated, are crucial for infection prevention. Antimicrobial prophylaxis is only one element integrating these interventions and should be limited to the period immediately after the trauma.

One major drawback of exposure to antibiotics is the emergence of drug-resistant bacteria strains, as the result of selection pressure on resistant colonizing strains or on bacteria with newly acquired mutations. Unfortunately, most studies dealing with antibiotic prophylaxis have not adequately investigated this issue, which is particularly relevant in critical care settings where bacterial resistance to antibiotics has become a major problem. Here, we focused on studies comparing antibiotic prophylaxis and placebo in reducing infections in several trauma-related conditions taking into account the risk of antibiotic-resistant bacteria selection to produce a fair risk/benefit ratio assessment, in contrast with other guidelines addressing similar issues [1], [2].

In the present paper, we report the contents of the review, the analysis and discussion of the literature, and the conclusions of the conference.

## Methods

The works was organized in three sequential steps:

*Step 1*: The Coordinating Committee formed by five intensivists selected an initial list of topics. Head, thorax, abdomen, and limbs trauma were considered. Because of time constraints (there were only two days to discuss the issues during the convention) only four clinical conditions were selected based on traumatic event frequency and/or infection severity. The scenarios included antibiotic prophylaxis for 1) early VAP in comatose patients, 2) meningitis in severe skull fractures, 3) wound infection in long-bone open fractures (we initially focused on osteomyelitis but found no study specifically investigating this issue), 4) deep surgical-site infections in abdominal trauma with hollow viscus perforation. We also considered but discarded pneumonia in thoracic contusion, empyema after chest tube insertion, surgical-site infection for long-bone closed fractures, infection after soft tissues open injuries, abdominal trauma without bowel perforation, and inhalation pneumonia in pre-hospital intubation.

The Coordinating Committee then generated the queries for the literature search and selected the articles fulfilling the criteria established for each subject. The fundamental criterion, shared by all the investigated issues, was the comparison between adult patients subjected to antibiotic prophylaxis and controls. When such studies were not available comparison between groups receiving different pharmacological protocols were considered.

The MEDLINE database was searched using the free PubMed provider. Randomised trials, reviews, meta-analyses, or observational studies were selected. Pediatric and animal studies, and articles not written in English, were excluded. The MEDLINE search covered a period between January 1970 and January 2014. This search was integrated with the consultation of reference lists of retrieved articles.

The Coordinating Committee members unanimously selected the articles after reviewing the abstracts, with the help of one of the authors with specific expertise in statistics and research methodology. Full-texts were then retrieved and a further selection was carried out.

The statistical and methodological expert also performed data extraction according to a predefined plan (the protocol was not pre-registered), using a dedicated electronic form that automatically performed all planed computations and plot generation. The electronic form was developed *ad hoc* for this revision, and tested and refined using the studies on early VAP prevention with antibiotic prophylaxis.

Data collected included study inclusion criteria, antibiotic class and dose, duration of prophylaxis, antibiotic-resistant bacteria selection when investigated, infection rates, and mortality rates when available, along with information needed for the GRADE evidence quality assessment.

Data were presented as event rates in treatment arms and controls, absolute risks, absolute risk reductions, and relative risks. Adjusted odds ratios were reported when dealing with multivariate analysis results in observational studies. Numbers needed to treat were also calculated. Confidence intervals were computed for all the above measures. Relative risks were graphically represented in Forest plots.

Design heterogeneity among selected studies advised against their combination in meta-analyses. For the same reason statistical approaches to investigate presence of asymmetry and potential publication bias were not carried out [3].

The queries and the results of the summarised results were submitted to the External Scientific Advisory Panel, formed by four intensivists with expertise in the field of infections and trauma in critical care settings, who discussed them together with Coordinating Committee members.

Evidence provided by randomised controlled trials (RCTs) and observational studies was ranked on the basis of the Grading of Recommendations Assessment, Development and Evaluation (GRADE) criteria. [4]–[6] The GRADE provides a framework to guide reviewers for the assessment of the strength of evidence, taking into account that different reviewers or reviewing groups can evaluate the same evidence differently. [7] Moreover, it eliminates automatic links between the strength of the recommendation and the strength of the evidence, leaving to the reviewers the possibility of grading recommendations based on other variables relevant to clinical decisions, besides evidence.

The GRADE stratifies quality of evidence on a four-level scale, ranging from high to very low, [7], in favour or against intervention. In the middle of this scale an absence of evidence level was added. This event occurs frequently in studies with small sample size, where the alternative hypothesis and statistical power have not been adequately taken into account. In our opinion, negative results from such studies should thus not be considered indicative of no effect, but rather of an unresolved issue [8].

The reporting of RCTs was evaluated on the basis of currently shared standards. [9] We considered quasi-randomised studies, in which treatment allocation is based on non-random methods such as alternation, leading to severe bias in regards to sequence generation and allocation concealment.

Our evaluation of meta-analyses was restrictive, according to the principle that heterogeneity among studies in terms of inclusion criteria, study design, study treatment, type of outcome, which are not accounted for by statistical methods focused on heterogeneity of outcome rates, [10] should contraindicate the merging of their results. [11] When dealing with observational studies we discarded al investigations that reported only crude-data comparisons without performing adjustment for potential confounders.

*Step 2*: The results were presented to the physicians invited to the symposium - intensivists, infectious disease specialists, surgeons, emergency physicians, radiologists, and physiatrists – and the issues were collectively discussed. The discussion was audio-recorded and at a later stage revised by members of the Coordinating Committee to identify and highlight the main opinions that emerged from the discussion.

*Step 3*: the scientific advisors and the Coordinating Committee members formed the jury that provided recommendations that were presented as preliminary results on the final day of the conference, but discussed and refined in the following months by e-mail. A final meeting was held one year after the conference, to discuss diverging opinions until unanimity was reached. To develop the recommendations the panel followed the GRADE framework. This is based on four points: quality of evidence, balance between desirable and undesirable effects, values and preferences, uncertainty about whether the intervention represents a wise use of resources [12].

The GRADE initially attributes a high default level of evidence to RCTs and a low level to observational studies. [7] RCTs can then be up- or downgraded according to the following criteria: study limitations due to insufficient internal validity, indirectness of evidence, imprecision of results, inconsistency between studies, and selective publication of studies. [13]–[17] While the first three are related to single studies, the last two points take into account the relation with other studies in terms of inconsistent results and publication bias, respectively. We added an *other* criterion which included other weak features of the study, such as the unbalance in important variables between study arms in RCTs that may occur when the sample size is small.

For RCTs the first criterion, i.e. bias, takes into account allocation concealment and blinding, completeness of follow-up and adherence to the intention-to-treat principle, premature study interruptions for benefit, and selective reporting of outcomes. [17] The last criterion takes into account the existence and availability of a study protocol or on the clear omission to report important outcomes. [17] We were not able to comply with this criterion because, for most RCTs, protocols were not available. For each of these items, concerns can be serious or very serious, down-scoring the evidence by one and two points respectively. For example, a RCT with an initial high-evidence grading can be downgraded to moderate or low, or even very low, if there are other weak points.

Limits in observational studies can include lack of controls, as in case series, or, when controls are present, lack of adjustment for important covariates, which may be unevenly distributed between study groups [17].

Initially, evidence is rated only one point up when the relative risk is between 0.5 and 0.2 or between 2 and 5, and two points up when lower than 0.2 or higher than 5, obviously when dealing with statistically significant results. However, even in the presence of large effects, when imprecision is strong (i.e. wide 95% confidence intervals) and negligible effects cannot be ruled out, upgrading is not performed. The rating can also be increased when a dose-response is evident or plausible potential confounders would only increase the confidence in the results [7], [18].

The strength of recommendations can be strong or weak, in favour or against interventions. [19] Only when an intervention with a strong likelihood of effectiveness had not been sufficiently investigated (i.e. absence of evidence as defined above) we considered the possibility of not giving any recommendation, although we usually gave a weak negative recommendation in most cases of absence of evidence, in the light of the limited resources available.

The conference dealt with four different issues of broad clinical interest but focusing especially on relevance for critically ill patients and from the point of view of intensive care unit (ICU) and emergency department physicians.

To investigate the main undesirable effects of prophylaxis, we preliminarily performed a systematic research on the effect of short antibiotic administration on bacterial antibiotic-resistance selection, to provide a fair assessment of the balance between benefits and risks. The results of this investigation integrated, when appropriate, the evidence concerning antibiotic prophylaxis in the clinical conditions we explored.

A transparent report of the reviewing process and of evidence grading is provided for each query in the supporting information.

The review was conducted complying with the Preferred Reporting Items for Systematic Reviews and Meta-Analyses (PRISMA) statement recommendations (Table S6 in File S6) [20].

## Results

We tried to find an answer to the following question: *Does short antibiotic prophylaxis cause resistant bacteria selection?* To do this we explored the influence of short antibiotic course on resistant bacteria selection at two levels: at the patient’s level, investigated before and after exposure, and at the centre level, comparing restrictive and liberal antibiotic prescription strategies between centres or within a single centre before and after the policy change.

We also assumed that there is strong evidence that prolonged antibiotic exposure (say, 7–10 days) increases the risk of colonization with resistant bacterial strains, [21] and without formally investigating this issue with the GRADE approach, we attributed to it a default high level of evidence.

### Selection of antibiotic-resistant bacteria in patients exposed to prophylaxis

The contemporaneous combination of the mutant bacteria and antibiotic exposure determines the spread of antibiotic-resistant bacteria. [22] Although patients admitted to the hospital after trauma are usually young and healthy, given the presence of resistant bacteria also within the community, the possibility that they may be already colonized by such strains cannot be ruled out. [23], [24] Moreover, colonization in non-colonized patients can occur because of newly acquired bacterial mutations, selection of bacterial resistant strains among heterogeneous populations, [25] and horizontal transfer, [26] the latter being particularly relevant in ICU environments. [27] We made an extensive literature revision focusing on antibiotic-resistant colonization in patients undergoing short antibiotic treatment, but RCTs in this field were unavailable. Thus, we had to rely on the few observational studies found (Table S5 in File S5).

In a study carried out on a cohort of 2,641 patients receiving antibiotic prophylaxis for cardiac surgery, of 1,094 undergoing cultures 426 were shown to harbour bacteria resistant to the antibiotic they were exposed to. Logistic regression, adjusting for many possible confounders, indicated an increased risk of resistance selection in case of antibiotic prophylaxis lasting more than 48 hours. [28] Overall the description of the statistical procedures was scanty, hindering quality assessment. One major limitation was that only part of the patients were submitted to cultures and that physicians in the centre were “encouraged” to keep antibiotic prophylaxis duration within 48 hours, potentially introducing selection biases that logistic regression may have not accounted for. Moreover, colonization status was not assessed before prophylaxis was administered, so it is not known whether the patients had previous colonization or acquired the resistant pattern during therapy or during the postoperative stay in a high-risk environment as the ICU.

In a before/after study involving 5 wards, the rate of acquisition within 30 days from initial antibiotic exposure, was 3% for MRSA (22/724), 2% for vancomycin-resistant enterococci (VRE) (10/430), and 1% (11/840) for ciprofloxacin-resistant *Pseudomonas Aeruginosa* (CRPA). [29] Forty per cent of newly acquired colonization with resistant bacteria was detected 48 hours from the first antibiotic dose. The initial colonization rate was 16% of 864 patients for MRSA, 50% for VRE, and 3% for ciprofloxacin-resistant *Pseudomonas Aeruginosa*. Cephalosporins, quinolones, and macrolides were the antibiotics more frequently administered (31, 29, 19% of cases, respectively). Absence of control (i.e. patient not exposed to antibiotics) although not irrelevant was not a major issue, in our opinion, because we do not expect factors other than antibiotic pressure to affect the colonization rate within a 48-hour period. A low colonization rate, as in this study, is still clinically relevant if we account for the duration and possibility of horizontal spread of resistant bacterial colonization, especially in environments such as the ICU [27], [30].

Another study investigated 29 patients undergoing cardiac surgery and 10 coronary angioplasty, receiving prophylaxis for three days with a first or a second-generation cephalosporin. [31] Cultures were collected at time 0 and on the third day. The investigators detected colonization by methicillin-resistant coagulase-negative staphylococci in 36 of 43 cultured sites (84%) previously not colonized and a quantitative colonization increase in 17 of 28 sites (61%) harbouring low-level colonization before antibiotic administration. The main limitation of the study was that multiple samples were collected from each patient that may have borne the same resistant bacteria, inflating the final count of true events. A reliable quantitative estimation of resistant bacteria selection was hence impossible. This study was also limited by its small sample size.

Finally, in a study investigating the effect of a five-day course with clarithromycin, 5 patients colonised by susceptible enterococcus strains few days after the end of treatment developed resistant types, which in 3 cases persisted for one year or more. [32] Although, almost a case report, this study is indicative of how antibiotic pressure can select resistant strains and how long the effects can last over time.

### Effect of liberal vs. aggressive antibiotic prescriptive strategies

The limitation of antibiotics use is widely recognized as an effective strategy for the reduction in bacterial resistance. [33], [34] However, the role of antibiotic prophylaxis policies, corresponding to strong limitations in antibiotic use, have never been investigated in relation to antibiotic-resistance acquisition. Indeed a recent review concerning the influence of antibiotic exposure on MRSA diffusion, the shortest period of antibiotic administration considered was 7 days. [21] In a before/after study dealing with late-generation cephalosporin administration policy, surgical prophylaxis was among the exceptions to restriction, indicating that the authors did not feel it was significant for the purposes of their policy [35].

*Conclusions:* there is low evidence indicating that antibiotic prophylaxis lasting 48 hours or more is capable of determining the colonization with resistant bacterial strains, that is the *undesirable effect* reported in the text and tables for each of the interventions under scrutiny.

### Question 1: is antibiotic prophylaxis for the prevention of ventilator-associated lower respiratory tract infections effective in intubated patients with traumatic head injury (TBI) and coma, i.e. Glasgow Coma Scale (GCS) ≤8?

*Results:* the MEDLINE search provided only three studies, two RCTs and one observational study, [36]–[38] all complying with our inclusion criteria. Although, the studies did not deal exclusively with TBI patients, the results can be reasonably extended to this category of patients. The two RCTs are described in Table 1, Table S1 in File S1, and Figure S1 and tested respectively a 3-day and a single-day antibiotic prophylaxis. Both RCTs reported a statistically significant reduction of early-VAP, but no reduction of late-onset pneumonia and ICU mortality. Besides other flaws, major limitations of both RCTs were the absence of blinding, the small sample size and the lack of power to detect increases in antibiotic-resistant infections in the treatment group (outcome investigated only in the first RCT). [36] Moreover, the second RCT was not specifically focused on comatose patients. [37] The premature interruption of the first RCT after the first interim analysis when only 38 patients were randomised, on the basis of the reduction of early-onset pneumonia incidence in the treatment arm, while no difference in mortality was detected, is a questionable choice. Finally, the beneficial effect of the postponement of pneumonia in a phase of minor vulnerability of the brain in severe brain injury is only theoretical and was not demonstrated with a robust outcome, such as the long-term mortality/severe disability rate. The main undesirable effect, i.e. antibiotic-resistant bacteria selection, according to the evidence we collected, was likely to occur after three days of antibiotic administration, while probably irrelevant when only two doses are given.

**Table 1:** Antibiotic prophylaxis for early-VAP prevention in comatose patients: patients, interventions, and outcomes.

			Desirable effect		Undesirable effect		Desirable effect			
Sample	Treatment	Control	Outcome	Outcome quality	Outcome	Outcome quality	Treatment arm % (95%-CI)	Controls % (95%-CI)	Difference % (95%-CI)	RR (95%-CI)
Ventilated patients, cerebral haemorrhage, GCS ≤8	Ampicillin-sulbactam 3-day course - 19 patients	Noplacebo -19 patients	Early VAP	Weak, surrogate of death/severe disability	Antibiotic- resistantbacteria selection not Adequately investigated	Robust, Clinicallyrelevant for thepatient/crucial forthe health-careorganization	21.1 (8.5 to 43.3)	57.9 (36.3 to 76.9)	–36.8	0.36 (0.14 to 0.94)
Ventilated patients, cerebral haemorrhage, GCS ≤12	2-dosecefuroxime - 50patients	Noplacebo - 50 patients	Early VAP	Weak, surrogate of death/severe disability	Antibiotic- resistantbacteria selection not investigated	Robust, Clinically relevant for the patient/crucial for the health-care organization	16 (8.3 to 28.5)	36 (24.1 to 49.9)	–20 (–35.8 to −2.8)	0.44 (0.21 to 0.93)

The results of a recent observational study, including 129 patients suggested that a single dose of ceftriaxone was an independent predictor of reduced incidence of early-onset pneumonia. [38] The regression analysis was performed including a propensity score and the variable “antibiotic prophylaxis” in a regression model, but the number of outcomes (overall 15 cases of early-onset VAP) did not justify the inclusion of the two above variables according established rules. [39], [40] Moreover, the sample size and the number of events were small, and the model was underpowered to include potential important covariates capable of influencing the significance of ceftriaxone administration in the predictive model. The observational study appeared to be, hence, unreliable and was not taken into account in our final evaluation.

*Conclusions:* The two RCTs had substantial different interventions and outcomes, thus we provided recommendations for two different queries (Table 2).

**Table 2:** Antibiotic prophylaxis for early-VAP prevention in comatose patients - Level of evidence and recommendations.

Patients and intervention	Desirable effect	Undesirable effect*	Benefit/risk profile	Values and preferences	Resource use	Recommendation	Rationale
Ventilated patients,cerebral haemorrhage,GCS≤8 –Ampicillin-sulbactam3-day course	Very lowevidence infavour ofintervention 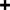 □□□	Lowevidenceagainstintervention 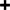 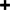 □□	Unfavourable	Not available	Unwise	Weak against intervention	The benefit/risk ratio was unfavourable. Resistant bacteria selection was considered a likely event for a three-day antibiotic course, a more robust and thus prevalent outcome compared to early-VAP reduction, a surrogate of middle term death and severe disability.
Ventilated patients,cerebral haemorrhage,GCS≤8-2-dosecefuroxime	Very lowevidence infavour ofintervention 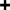 □□□	No evidenceagainstintervention□□□□	Uncertain	Not available	Not assessable	Weak against intervention	The intervention requires further investigation focused on hard outcomes such as death and severe disability. The selection of resistant bacteria appears to be unlikely with two doses of antibiotic although it cannot be ruled out. Notwithstanding the large effect size (RR 0.44), given the high degree of imprecision a limited reduction of early VAP (lower 95%-CI = 0.93) is possible, corresponding to a 2.8 absolute risk reduction and a NNTB of 36, a small effect at the price of an excessively high number of patients to expose to antibiotics, especially in the context of the ICU.

*Not investigated in the studies, evidence could be found from external sources. NNTB  = number needed to treat for benefit.

#### Intervention 1

Three-day course with a beta-lactamase-protected penicillin for the prevention of ventilator-associated early-VAP in intubated patients with TBI and coma:

Level of evidence in favour: Very low evidence.
Level of evidence against: Low evidence.
Recommendation: Weak against intervention.

#### Intervention 2

2-dose wide spectrum antibiotic administration for the prevention of early-VAP in intubated patients with TBI and coma.

Level of evidence in favour: Very low evidence.
Level of evidence against: No evidence.
Recommendation: Weak against intervention.

### Question 2: is antibiotic prophylaxis effective in patients with basilar skull fractures from non-penetrating head trauma to decrease the occurrence of meningitis?

*Results:* of 146 studies found only four were RCTs. One was discarded because only 10 patients were randomised while 129 were the object of a retrospective evaluation and therefore included among observational studies. [41] The remaining were single-centre trials, including 109, 37, and 52 patients respectively, all providing negative results, but the three studies were underpowered to demonstrate clinically relevant differences (Table 3, Table S2 in File S2, Figure S2). [42]–[44] The 37 patients enrolled in the second study with basilar fractures were a subset of 196 with either basilar or skull open fractures. Thus the findings of interest for our review were the result of a subgroup analysis, and as such biased. [45] The other two trials also suffered from methodological bias. Most important long-lasting antibiotic prophylaxis was administered (5-day ceftriaxone, 3-day ceftriaxone or ampicillin-sulbactam, and 8-day average penicillin course, respectively) and no monitoring of antibiotic-resistant bacteria selection was investigated. This was a major undesirable effect of such prolonged antibiotic prophylaxis, as supported by the literature revision we carried out.

**Table 3:** Antibiotic prophylaxis for the prevention of meningitis in basilar skull fractures: patients, interventions, and outcomes. NC = not computable.

			Desirable effect		Undesirable effect		Desirable effect			
Sample	Treatment	Control	Outcome	Outcome quality	Outcome	Outcome quality	Treatment arm % (95%-CI)	controls % (95%-CI)	Difference % (95%-CI)	RR (95%-CI)
Open skullfractures or basilar skullfractures	3-day courseceftriaxone or ampicillin-sulphadiazine - 25 patients	No placebo - 12 patients	Meningitis reduction	Robust, Prognostically relevant outcome	Antibiotic- resistant bacteria selectionnot investigated	Robust, Clinically relevant for the patient/crucial for the health-care organization	0 (0 to 13.3)	8.3 (1.5 to 35.4)	-8.3 (-35.4 to 6.6)	NC
Acutetraumatic pneumo-cephalusverified by CT scan	5-day course ceftriaxone- 53 patients	No placebo - 56 patients	Meningitis reduction	Robust, Prognostically relevant outcome	Antibiotic- resistant bacteria selectionnot investigated	Robust, Clinically relevant for the patient/crucial for the health-care organization	18.9 (10.6 to 31.4)	21.4 (12.7 to 33.8)	-2.6 (-17.5 to 12.7)	0.88 (0.42 to 1.86)
Traumatic rhinorrhoea orotorrhoea	Average 7.7 days course penicillin - 26 patients	Placebo - 26 patients	Meningitis reduction	Robust, Prognostically relevant outcome	Antibiotic- resistant bacteria selection not investigated	Robust, Clinically relevant for the patient/crucial for the health-care organization	0 (0 to 12.9)	3.8 (0.7 to 18.9)	-3.8 (-18.9 to 9.4)	NC

The negative evidence regarding this issue are summarised in a recent Cochrane review and meta-analysis [46] that also includes the study of the randomised ten patients mentioned above and a RCT published as correspondence. [47] Although the studies were not heterogeneous in terms of meningitis odds ratios, they were heterogeneous in terms of meningitis absolute rates, inclusion criteria, duration of antibiotic treatment, ceftriaxone dose in the two studies that used this antibiotic, [43], [44] and number of patients lost to follow-up, advising against their inclusion in a meta-analysis. Two other non-recent meta-analyses were biased by the merging of RCTs and observational studies [48], [49].

We found four observational studies matching the inclusion criteria, none of which performed adjusted comparisons, thus not providing any acceptable evidence in favour of or against antibiotic prophylaxis [41], [50]–[52].

*Conclusions:* The three studies under scrutiny all investigated prolonged antibiotic administration, that was thus the object of our query (Table 4).

**Table 4:** Antibiotic prophylaxis for the prevention of meningitis in basilar skull fractures - Level of evidence and recommendations.

Patients and intervention	Desirable effect	Undesirable effect*	Benefit/risk profile	Values and preferences	Resource use	Recommendation	Rationale
Open skull fractures or basilar skull fractures - 3-day course ceftriaxone or ampicillin/sulphadiazine	No evidence infavour of intervention □□□□	Low evidenceagainst intervention 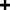 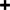 □□	Unfavourable	Not available	Unwise	Strong against intervention	
Acute traumatic pneumocephalus verified by CT scan - 5-day course ceftriaxone	No evidence infavour of intervention □□□□	Low evidenceagainst intervention 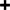 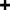 □□	Unfavourable	Not available	Unwise	Strong against intervention	Results of the RCTs were negative. The studies were seriously biased The risk of resistant bacteria selection is a likely and relevant undesirable effect when prolonged antibiotic prophylaxis is administered.
Traumatic rhinorrhoea or otorrhoea - Average 7.7 days course penicillin	No evidence in favour of intervention □□□□	Low evidence against intervention 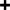 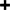 □□	Unfavourable	Not available	Unwise	Strong against intervention	

*Not investigated in the studies, evidence could be found from external sources.

#### Intervention

Three or more days of antibiotic prophylaxis for the prevention of meningitis in patients with basal-skull fractures:

Level of evidence in favour: No evidence.
Level of evidence against: Low evidence.
Recommendation: Strong against intervention.

### Question 3: is antibiotic prophylaxis beneficial for the reduction of wound infection rate in patients with long-bone open fractures?

*Results:* of 174 studies fulfilling the search criteria, only three trials, [53]–[55] one observational study, [56] and one meta-analysis [57] matched the inclusion criteria. The studies were carried out in between the 1970s and the 1980s. In the first RCT, patients with open-fractures were only a subset of the randomised sample, [53] the findings thus being the result of a subgroup analysis and as such inadequate to provide conclusive answers. [45] The second study [54] was of higher level, but still the reporting was insufficient to allow a thorough assessment of its quality according to current standards. [9] In these two studies, the diagnosis required positive cultures. The third study was very low quality, with no blinding and no placebo, and a partial report of the results. It was carried out on open fractures due to gunshot, with infections diagnosis not based on cultures. [55] This study was negative, since 2 cases out of 32 were reported in the single-day cefazolin group and 2 out of 35 in the group not receiving antibiotic. We considered this study methodologically too weak to be considered a reliable source of evidence.

The antibiotic tested in the first two RCTs (Table 5, Table S3 in File S3, Figure S3) were instead dicloxacillin or benzyl penicillin, [53] and cloxacillin, [54] respectively. In the first study treatment was continued for 48 hours and, in the second, for ten days. The influence of prophylaxis on bacterial resistance to antibiotics was not investigated in any of these studies. We assumed that a 10-day antibiotic administration would certainly greatly increase the risk of antibiotic-resistant bacteria selection. We attributed, instead, a lower level of evidence to a 48-hour treatment (Table 6).

**Table 5:** Antibiotic prophylaxis for the prevention of wound infections in long-bone open fractures: patients, interventions, and outcomes.

			Desirable effect		Undesirable effect		Desirable effect			
Sample	Treatment	Control	Outcome	Outcome quality	Outcome	Outcome quality	Treatment arm % (95%-CI)	Controls % (95%-CI)	Difference %(95%-CI)	RR (95%-CI)
Long-bone open fractures	48-hour course, dicoxacillin or penicillin - 60 patients	Placebo - 30 patients	Wound infection (not specifically osteomyelitis)	Weak, Minor prognostic relevance	Antibiotic- resistant bacteria selection, not investigated by the study	Robust, Clinically relevant for the patient/crucial for the health-care organization	6.7 (2.6 to 15.9)	20 (9.5 to 37.3)	–13.3 (–31.1 to 0.7)	0.33 (0.1 to 1.09)
Long-bone open fractures	cloxacillin 10-day course - 43 patients	Placebo - 44 patients	Wound infection (not specifically osteomyelitis)	Weak, Minor prognostic relevance	Antibiotic- resistant bacteria selection, not investigated by the study	Robust, Clinically relevant for the patient/crucial for the health-care organization	4.7 (1.3 to 15.5)	27.3 (16.3 to 41.8)	–22.6	0.17 (0.04 to 0.72)

**Table 6:** Antibiotic prophylaxis for the prevention of wound infections in long-bone open fractures - Level of evidence and recommendations.

Patients and intervention	Desirable effect	Undesirable effect*	Benefit/risk profile	Values and preferences	Resource use	Recommendation	Rationale
Long-bone openfractures - 48-hour course dicoxacillin or penicillin	No evidencein favourof intervention □□□□	Low evidenceagainstintervention 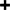 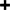 □□	Unfavourable	Notavailable	Unwise	Weak againstintervention	The study was slightly negative but the power was insufficient to detect clinically significant differences. Being the result of a subgroup analysis the effectiveness of the intervention is only hypothetical. There is evidence of risk for resistant bacteria selection even with a two-day antibiotic course. Wound infections (excluding osteomyelitis) are not as relevant an outcome as resistant bacteria selection, given the potentially high number needed to treat (Table S3 in File S3).
Long-bone openfractures - 10-daycourse cloxacillin	Low evidencein favourof intervention 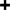 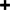 □□	High evidenceagainstintervention 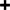 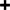 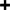 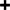	Unfavourable	Notavailable	Unwise	Strong againstintervention	It is common knowledge that 10-day antibiotic prophylaxis has a very high chance of determining resistant bacteria selection, which outweighs wound. infections prevention, generating an unfavourable benefit/risk profile.

*Not investigated in the studies, evidence could be found from external sources.

A 13.3 and 22.6% wound-infection rate decrease was found in the antibiotic-prophylaxis arms in the two studies, corresponding respectively to an almost statistically significant result and a fully statistically significant result. Interestingly, in the second study, infections occurring within six weeks were considered early infections, including infections that, reasonably, should have not been influenced by early antibiotic prophylaxis. In the first study, instead, the duration of the “window” period was not specified.

The observational study that matched the inclusion criteria suggested a protective effect of antibiotic prophylaxis, but since it was based only on the crude infection rate, it was not included in the analysis [56].

The meta-analysis included, besides the three RCT mentioned above, two other studies that we excluded because one was written in German [58] and the other including hand, foot, and fingers fracture, pediatric patients and gunshot injuries, without performing any subgroup analysis on long-bone fractures. [59] The meta-analysis concluded that antibiotic prophylaxis is effective in reducing wound infections. However, study designs, inclusion criteria, treatment protocols, and quality of the trials, were so heterogeneous to question the reliability of their merging in a single sample. We hence considered this study a potential source of misleading information.

We did not find any study comparing no antibiotic prophylaxis with 24-hours antibiotic prophylaxis, an option recommended by other guidelines presumably on the basis of indirect evidence [1], [2], [60], [61].

*Conclusions:* Two different antibiotic strategies were adopted in the two studies that were included in the final evaluation, leading to two answers to two different queries (Table 6).

#### Intervention 1

2-day antibiotic administration for the prophylaxis of wound infections (not specifically osteomyelitis).

Level of evidence in favour: No evidence.
Level of evidence against: Low evidence.
Recommendation: Weak against intervention.

#### Intervention 2

Prolonged (ten-day) antibiotic prophylaxis for the prevention of wound infections (not specifically osteomyelitis).

Level of evidence in favour: Low evidence.
Level of evidence against: High evidence.
Recommendation: Strong against intervention.

### Question 4: is antibiotic prophylaxis indicated to reduce the risk of deep surgical site infections in patients with abdominal trauma and enteric abdominal contamination submitted to emergent surgery?

*Results:* out of 504 articles resulting from the literature search, none compared treatment and no-treatment arms. Our results were consistent with those of two recent Cochrane meta-analyses that did not find any RCTs investigating the effectiveness of antibiotic prophylaxis compared to placebo in reducing infections following penetrating abdominal trauma [62], [63].

Since no direct evidence exists with regard to the effectiveness of antibiotic prophylaxis in abdominal trauma compared to placebo, under the assumption that antibiotic prophylaxis is effective in preventing surgical site infections, [64] we oriented the query towards the duration of antibiotic prophylaxis as it was studied by three RCTs focused on penetrating abdominal trauma and deep surgical infections occurrence, [65]–[67] and excluded a fourth one because it did not report the rate of deep surgical site infections. [68] The main limitation of the studies was that only a minority of the patients had associated hollow viscus injury. Moreover, the infection rate for those who bore intestinal perforations was not reported, making it impossible to analyse subgroup performance. Thus, it was only possible to study the number of events in relation to the overall sample, including those without perforation. Another weakness was that no evidence concerning blunt trauma, a common event in abdominal trauma, was available.

The first two studies dealt almost exclusively with deep surgical site infections, i.e. intra-abdominal abscess, peritonitis, and necrotizing fasciitis, while the third one reported disaggregated data concerning deep and superficial surgical site infections. Thus, we were able to analyse data concerning deep surgical infections. The studies recruited 317, 515, and 300 patients respectively, and infections rates were 10 vs. 8% in the 5-day and 24-hour treatment arms in the first two studies, and 6% in both arms in the third one (Table 7). Although the studies were underpowered to detect clinically meaningful differences in the outcome rate (the power to detect a 5% difference ranged between 40 and 60% in the three RCTs), looking at the range of the absolute-difference 95%-confidence intervals, there is little chance that a clinically relevant superiority of 5-day prophylaxis is present (Table 7, Table S4 in File S4, Figure S4). Moreover, on the basis of our specific evidence assessment, five days of antibiotic administration have a high probability of determining resistant-bacterial selection unlike a 24-hour therapy.

**Table 7:** Antibiotic prophylaxis for the prevention of deep surgical-site infections in abdominal trauma with enteric contamination: patients, interventions, and outcomes.

			Desirable effect		Undesirable effect		Desirable effect			
Sample	Treatment	Control	Outcome	Outcome quality	Outcome	Outcome quality	Treatment arm % (95%-CI)	Controls % (95%-CI)	Difference % (95%-CI)	RR (95%-CI)
Penetrating abdominal trauma	24-hourcefoxitin or cefotetan -265 patients	5-day cefoxitin or cefotetan - 250 patients	Deep surgical site infections	Robust, Prognosticallyrelevant outcome	Antibiotic-resistant bacteria selection, not investigated in the study	Robust, Clinically relevant for the patient/crucial for the health-care organization	7.9 (5.2 to 11.8)	10 (6.9 to 14.3)	–2.1	0.79 (0.46 to 1.38)
Penetrating abdominal trauma	24-hourampicillin- sulbactam - 158 patients	5-day ampicillin- sulbactam - 159 patients	Deep surgical site infections	Robust, Prognosticallyrelevant outcome	Antibiotic-resistant bacteria selection, not investigated in the study	Robust, Clinically relevant for the patient/crucial for the health-care organization	8.2 (4.9 to 13.6)	10.1 (6.3 to 15.7)	–1.8	0.82 (0.41 to 1.64)
Penetrating abdominal trauma	24-hour cefoxitin - 148 patients	5-day cefoxitin - 152 patients	Deep surgical site infections	Robust, Prognostically relevant outcome	Antibiotic-resistant bacteria selection, not investigated in the study	Robust, Clinically relevant for the patient/crucial for the health-care organization	6.1 (3.2 to 11.2)	5.9 (3.1 to 10.9)	0.2 (–5.5 to 5.9)	1.03 (0.42 to 2.52)

In the studies, surgical management was carried out within few hours from the injury, and early surgery appears to be a reasonable condition for short (i.e. ≤24 hours) antibiotic prophylaxis. We adopted the Infective Disease Society of America (IDSA) definition of early surgery (i.e. carried out within 12 hours from trauma). [69] There is currently no clear proof in favour of this cut-off but it, nevertheless, seems reasonable.

*Conclusions:* The review was focused on different antibiotic strategies under the assumption that antibiotic prophylaxis is effective in reducing infections compared to placebo (Table 8).

**Table 8:** Antibiotic prophylaxis for the prevention of deep surgical-site infections in abdominal trauma with enteric contamination - Level of evidence and recommendations.

Query	Desirable effect	Undesirable effect*	Benefit/risk profile	Values and preferences	Resource use	Recommendation	Rationale
Penetrating abdominal trauma - 24-hour cefoxitin or cefotetan vs. 5-day cefoxitin or cefotetan	Very low evidence in favour of intervention 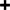 □□□	No evidence against intervention □□□□	Favourable	Not available	Wise	Weak in favour of intervention	Only part of the patients had intestinal perforation, but no specific data is available for this subset; this hampers conclusive answers regarding patients with perforation, the focus of the review. However, the risk of resistant bacteria selection is itself a sufficient
Penetrating abdominal trauma - 24-hour ampicillin-sulbactam vs. 5-day ampicillin-sulbactam	Very low evidence in favour of intervention 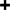 □□□	No evidence against intervention □□□□	Favourable	Not available	Wise	Weak in favour of intervention	condition to contraindicate the 5-day antibiotic course. We assume that single-day prophylaxis is effective on the basis of indirect evidence from elective abdominal-surgery antibiotic prophylaxis studies and less dangerous in terms of antibiotic-resistant bacteria selection than a 5-day treatment. This issue however deserves further investigations.
Penetrating abdominal trauma - 24-hour cefoxitin vs. 5-day cefoxitin	Very low evidence in favour of intervention 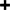 □□□	No evidence against intervention □□□□	Favourable	Not available	Wise	Weak in favour of intervention	

*Not investigated in the studies, no evidence could be found from external sources.

#### Intervention

Administration of 24-hour is equivalent to 5-day antibiotic prophylaxis for surgical abdominal trauma with hollow viscus injury and surgical repair within 12 hours of trauma:

Level of evidence in favour: Very low evidence.
Level of evidence against: No evidence.
Recommendation: Weak in favour intervention.

## Discussion

The main limitation of our study is that the literature search was limited to the MEDLINE database and only to publications in English, because of financial constraints. However, the issues we have dealt with have been largely investigated in previous reviews, and the guidelines included in the literature we have scrutinized wee used as an alternative source of references. We are therefore confident that we did not miss important publications in this field.

It is our firm belief that our recommendations, and guidelines in general, should not be clinical rules that impose specific clinical behaviours on doctors, but only indications applicable to an average patient, and should never replace the complex and individualised decisional process that physicians follow for individual patients [70].

In studying the role of antibiotic prophylaxis in trauma we found that in many cases the studies did not provide sufficient information for GRADE categories to be fully applied. Specifically, the assessment of the overall benefit/risk profile requires the comparison between desirable and undesirable effects. In the case of prolonged antibiotic prophylaxis (in several scrutinized studies antibiotic were administered for three days or more), the undesirable effect is the selection of antibiotic-resistant bacteria, an outcome not investigated in most of the studies. [71] Thus we had to integrate studies investigating exclusively infection prevention with evidence from other sources dealing with selection of resistant bacteria, a novel approach compared to other experiences that have proposed a different interpretation of evidence in this field [1], [2].

The risk of resistant mutant selection due to short antibiotic pressure has not been investigated with RCTs. We had therefore to rely on before-after studies investigating either the colonization of single patients after treatment or the effect of antibiotic policies changes in single centres.

We were able to find observational studies suggesting that a 2/3-day antibiotic course could already result in the selection of antibiotic-resistant bacteria. We graded this evidence as low or very low, due to several limitations of the studies. However, in the face of similar strength of evidence in favour and against the intervention, as in the case of the 3-day antibiotic prophylaxis for wound infections in open-fractures or early-VAP prevention in comatose patients, we considered the antibiotic-resistant resistant selection the main concern in terms of clinical impact and social relevance of the outcome., Antibiotic-resistant bacteria spread constitutes an emergency for the global health-care system, given its high burden of mortality and resource consume. [72]–[74] Since resistant mutations are widespread in hospitals and extending within the community, the solution to this problem appears to be the restoration of susceptible bacteria by strongly limiting antibiotic use. [22] Our concern is that the common use of antibiotics in prophylaxis, even for short periods, might significantly worsen this problem. Our recommendations go in this direction.

In conclusion, we would like to mention a striking report from a neurosurgical centre that in the late 1960s had to face an outbreak of resistant *Klebsiella Aerogenes*, which at its peak caused urinary infections in 1 of 4 patients, respiratory infections in 1 of 8, and 9 cases of meningitis 8 of which were fatal. [75]–[77] Though antibiotic treatment was targeted on sensitivity tests and despite the major increase in antibiotic consumption in the ward between 1966 ad 1969 the outbreak was out of control. As the authors state, “in desperation”, antibiotic use whether for treatment or prophylaxis was abandoned. This resulted in an immediate reduction of infections and the elimination of the Klebsiella from the ward without any serious adverse event being reported as the consequence of this strategy. This report although deviating from the paradigm of evidence base medicine (we are dealing with a single-centre epidemiological descriptive report with no statistical analysis performed and no precise design) nevertheless provides a demonstration of the effectiveness of a drastic antibiotic containment strategy, a potential choice when “the going gets tough”.

## Supporting Information

Figure S1 **Absolute proportions differences and relative risks for the studies concerning the first query.**
(PDF)

Figure S2 **Absolute proportions differences and relative risks for the studies concerning the second query.**
(PDF)

Figure S3 **Absolute proportions differences and relative risks for the studies concerning the third query.**
(PDF)

Figure S4 **Absolute proportions differences and relative risks for the studies concerning the fourth query.**
(PDF)

File S1 **MEDLINE database search, flow diagram illustrating the literature selection process, and Table S1 illustrating evidence assessment for the first query.**
(DOCX)

File S2 **MEDLINE database search, flow diagram illustrating the literature selection process, and Table S2 illustrating evidence assessment for the second query.**
(DOCX)

File S3 **MEDLINE database search, flow diagram illustrating the literature selection process, and Table S3 illustrating evidence assessment for the third query.**
(DOCX)

File S4 **MEDLINE database search, flow diagram illustrating the literature selection process, and Table S4 illustrating evidence assessment for the fourth query.**
(DOCX)

File S5 **MEDLINE database search, flow diagram illustrating the literature selection process, and Table S5 illustrating evidence assessment for antibiotic-resistant bacteria (undesirable effect).**
(DOCX)

File S6 **Preferred Reporting Items for Systematic Reviews and Meta-Analyses (PRISMA) statement recommendations checklist (Table S6).**
(DOC)
