# Supplementary figures and images for: Systematic Review of the Literature and Evidence-Based Recommendations for Antibiotic Prophylaxis in Trauma: Results from an Italian Consensus of Experts
Source: PLoS One. 2014 Nov 20;9(11):e113676. doi: 10.1371/journal.pone.0113676 (PMC4239082; doi:10.1371/journal.pone.0113676)

# RCTs - % difference between Controls and Treatment

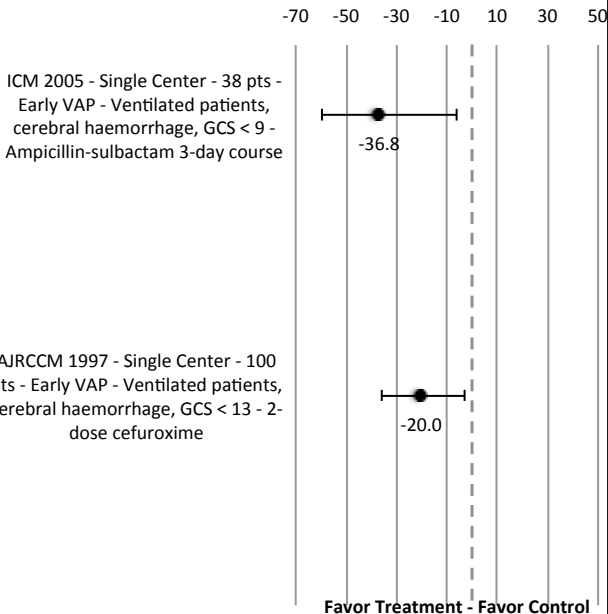

# RCTs - RR Treatment/Controls

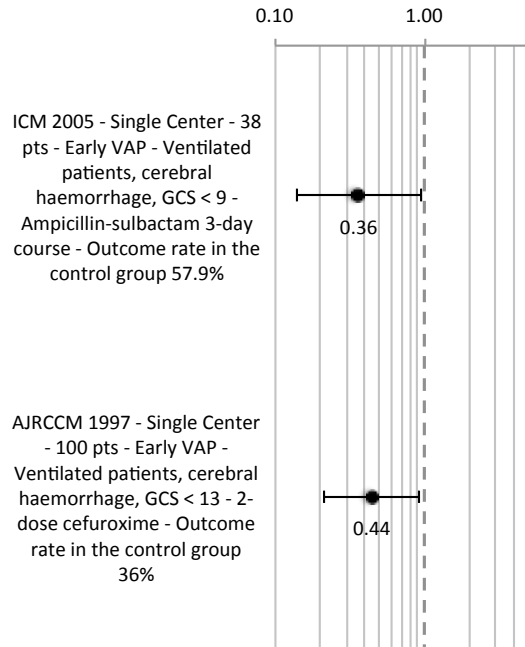

Supplement: Figure S1 — Absolute proportions differences and relative risks for the studies concerning the first query. (PDF) [file pone.0113676.s001.pdf]
