# Supplementary material for: Systematic Review of the Literature and Evidence-Based Recommendations for Antibiotic Prophylaxis in Trauma: Results from an Italian Consensus of Experts
Source: PLoS One. 2014 Nov 20;9(11):e113676. doi: 10.1371/journal.pone.0113676 (PMC4239082; doi:10.1371/journal.pone.0113676)
Supplement: Figure S2 — Absolute proportions differences and relative risks for the studies concerning the second query. (PDF) [file pone.0113676.s002.pdf]

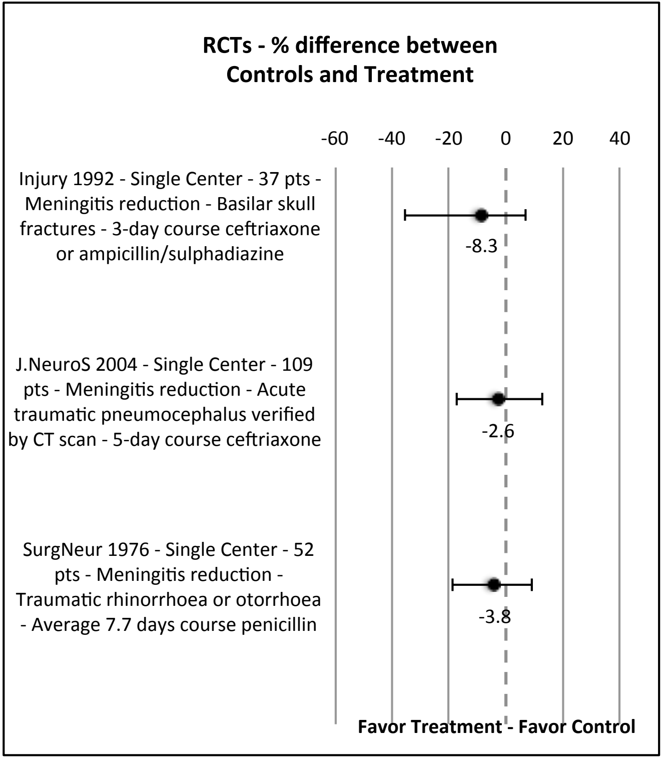
RCTs - % difference between Controls and Treatment
-60
-40
-20
0
20
40
Injury 1992 - Single Center - 37 pts - Meningitis reduction - Basilar skull fractures - 3-day course ceftriaxone or ampicillin/sulphadiazine
-8.3
J.NeuroS 2004 - Single Center - 109 pts - Meningitis reduction - Acute traumatic pneumocephalus verified by CT scan - 5-day course ceftriaxone
-2.6
SurgNeur 1976 - Single Center - 52 pts - Meningitis reduction - Traumatic rhinorrhoea or otorrhoea - Average 7.7 days course penicillin
-3.8
Favor Treatment - Favor Control

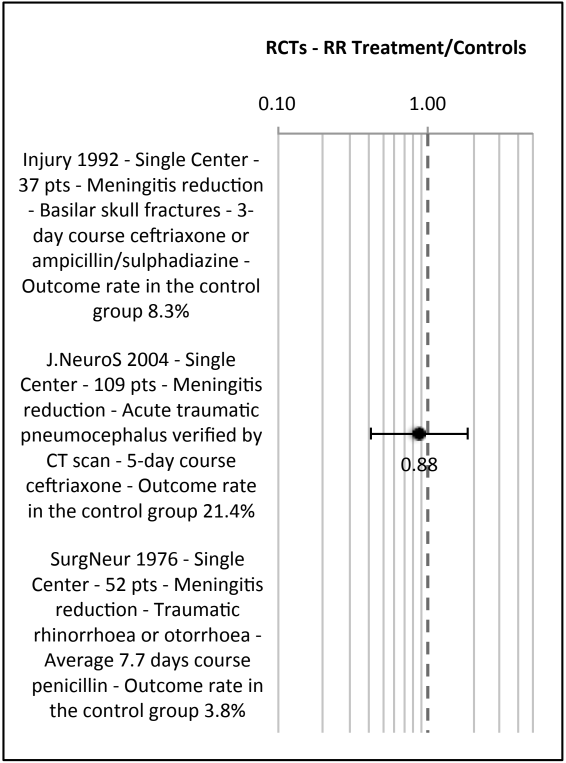
RCTs - RR Treatment/Controls
0.10
1.00
Injury 1992 - Single Center - 37 pts - Meningitis reduction - Basilar skull fractures - 3-day course ceftriaxone or ampicillin/sulphadiazine - Outcome rate in the control group 8.3%
J.NeuroS 2004 - Single Center - 109 pts - Meningitis reduction - Acute traumatic pneumocephalus verified by CT scan - 5-day course ceftriaxone - Outcome rate in the control group 21.4%
0.88
SurgNeur 1976 - Single Center - 52 pts - Meningitis reduction - Traumatic rhinorrhoea or otorrhoea - Average 7.7 days course penicillin - Outcome rate in the control group 3.8%
