# Supplementary material for: Systematic Review of the Literature and Evidence-Based Recommendations for Antibiotic Prophylaxis in Trauma: Results from an Italian Consensus of Experts
Source: PLoS One. 2014 Nov 20;9(11):e113676. doi: 10.1371/journal.pone.0113676 (PMC4239082; doi:10.1371/journal.pone.0113676)
Supplement: Figure S3 — Absolute proportions differences and relative risks for the studies concerning the third query. (PDF) [file pone.0113676.s003.pdf]

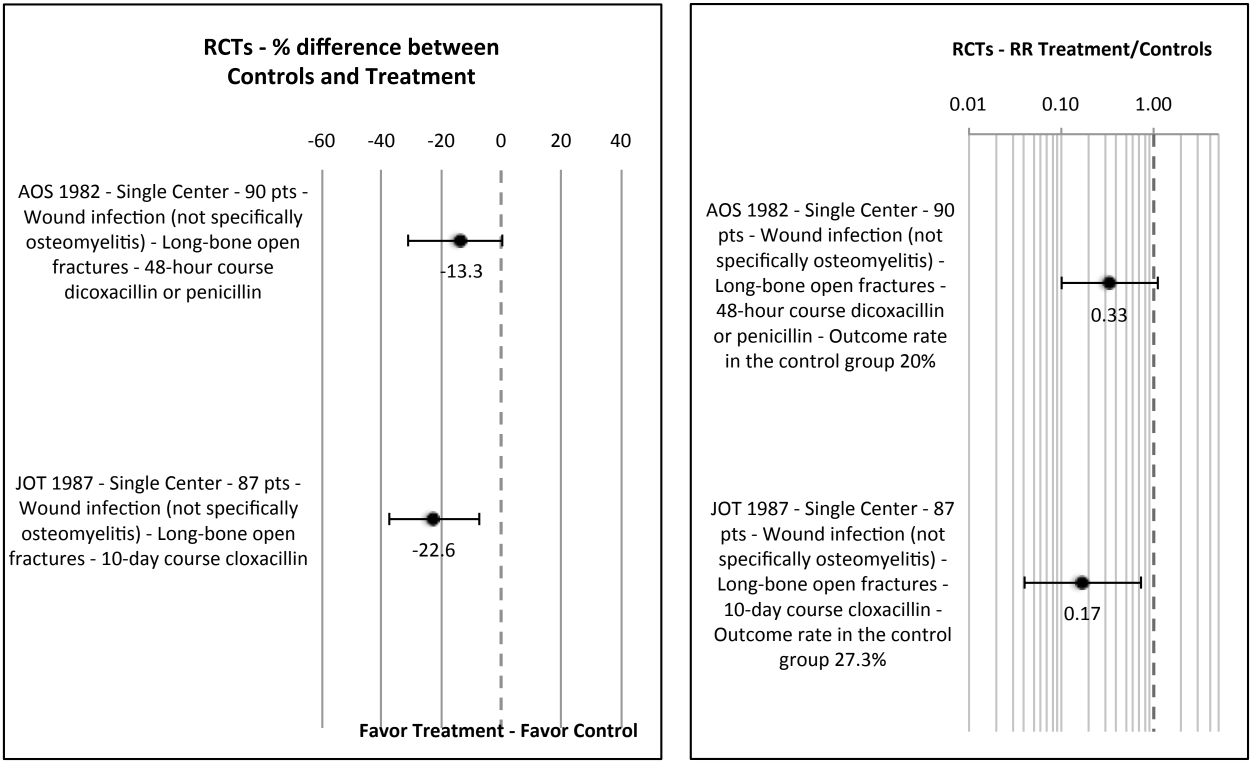
RCTs - % difference between Controls and Treatment
-60
-40
-20
0
20
40
AOS 1982 - Single Center - 90 pts - Wound infection (not specifically osteomyelitis) - Long-bone open fractures - 48-hour course dicoxacillin or penicillin
-13.3
JOT 1987 - Single Center - 87 pts - Wound infection (not specifically osteomyelitis) - Long-bone open fractures - 10-day course cloxacillin
-22.6
Favor Treatment - Favor Control
RCTs - RR Treatment/Controls
0.01
0.10
1.00
AOS 1982 - Single Center - 90 pts - Wound infection (not specifically osteomyelitis) - Long-bone open fractures - 48-hour course dicoxacillin or penicillin - Outcome rate in the control group 20%
0.33
JOT 1987 - Single Center - 87 pts - Wound infection (not specifically osteomyelitis) - Long-bone open fractures - 10-day course cloxacillin - Outcome rate in the control group 27.3%
0.17
