# Supplementary material for: Systematic Review of the Literature and Evidence-Based Recommendations for Antibiotic Prophylaxis in Trauma: Results from an Italian Consensus of Experts
Source: PLoS One. 2014 Nov 20;9(11):e113676. doi: 10.1371/journal.pone.0113676 (PMC4239082; doi:10.1371/journal.pone.0113676)
Supplement: Figure S4 — Absolute proportions differences and relative risks for the studies concerning the fourth query. (PDF) [file pone.0113676.s004.pdf]

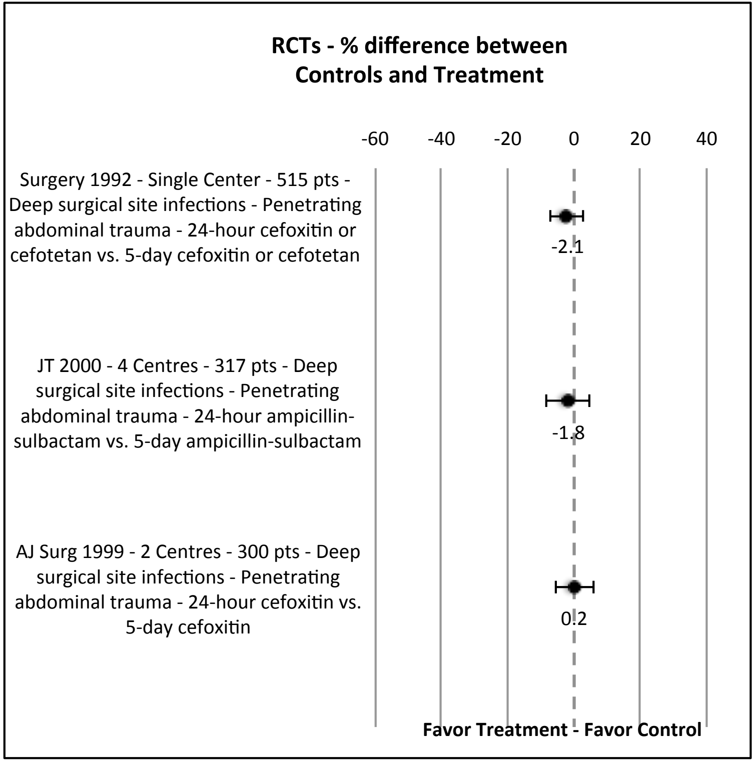
RCTs - % difference between Controls and Treatment
-60
-40
-20
0
20
40
Surgery 1992 - Single Center - 515 pts - Deep surgical site infections - Penetrating abdominal trauma - 24-hour cefoxitin or cefotetan vs. 5-day cefoxitin or cefotetan
-2.1
JT 2000 - 4 Centres - 317 pts - Deep surgical site infections - Penetrating abdominal trauma - 24-hour ampicillin-sulbactam vs. 5-day ampicillin-sulbactam
-1.8
AJ Surg 1999 - 2 Centres - 300 pts - Deep surgical site infections - Penetrating abdominal trauma - 24-hour cefoxitin vs. 5-day cefoxitin
0.2
Favor Treatment - Favor Control

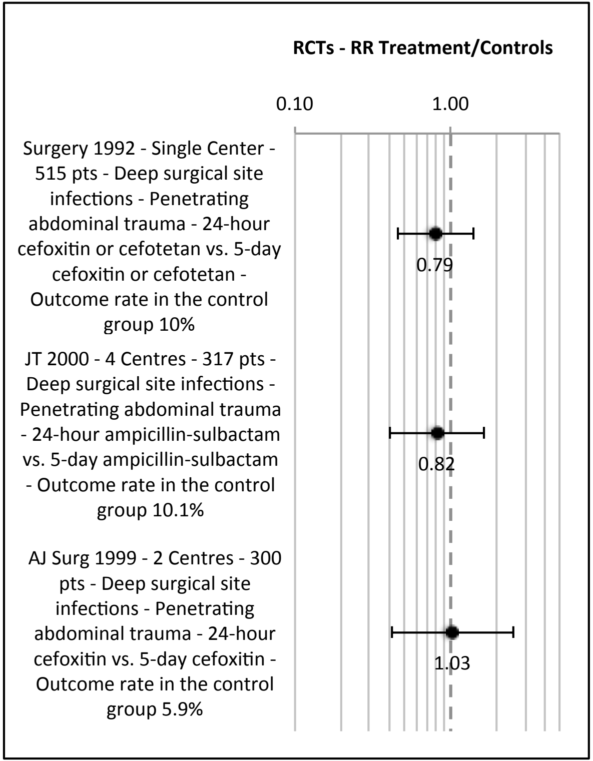
RCTs - RR Treatment/Controls
0.10
1.00
Surgery 1992 - Single Center - 515 pts - Deep surgical site infections - Penetrating abdominal trauma - 24-hour cefoxitin or cefotetan vs. 5-day cefoxitin or cefotetan - Outcome rate in the control group 10%
0.79
JT 2000 - 4 Centres - 317 pts - Deep surgical site infections - Penetrating abdominal trauma - 24-hour ampicillin-sulbactam vs. 5-day ampicillin-sulbactam - Outcome rate in the control group 10.1%
0.82
AJ Surg 1999 - 2 Centres - 300 pts - Deep surgical site infections - Penetrating abdominal trauma - 24-hour cefoxitin vs. 5-day cefoxitin - Outcome rate in the control group 5.9%
1.03
