# Supplementary material for: Systematic Review of the Literature and Evidence-Based Recommendations for Antibiotic Prophylaxis in Trauma: Results from an Italian Consensus of Experts
Source: PLoS One. 2014 Nov 20;9(11):e113676. doi: 10.1371/journal.pone.0113676 (PMC4239082; doi:10.1371/journal.pone.0113676)
Supplement: File S1 — MEDLINE database search, flow diagram illustrating the literature selection process, and Table S1 illustrating evidence assessment for the first query. (DOCX) [file pone.0113676.s005.docx]

**File S1:** MEDLINE database search, flow diagram illustrating the literature selection process, and evidence assessment for the first query in **table S1**.

**Question 1:** *is an antibiotic prophylaxis for the prevention of ventilator-associated lower respiratory tract infections effective in intubated patients with traumatic head injury (TBI) and coma, i.e. Glasgow Coma Scale (GCS) ≤8?*

**MEDLINE database search:** selection included clinical trials and observational studies, published after 1970 and written in English, comparing adults receiving antibiotic prophylaxis and control groups not receiving any antibacterial drug were selected.

**PubMed search details**

((("coma"[MeSH Terms] OR "coma"[All Fields]) AND ("antibiotic prophylaxis"[MeSH Terms] OR ("antibiotic"[All Fields] AND "prophylaxis"[All Fields]) OR "antibiotic prophylaxis"[All Fields])) AND (("pneumonia"[MeSH Terms] OR "pneumonia"[All Fields]) AND ("prevention and control"[Subheading] OR ("prevention"[All Fields] AND "control"[All Fields]) OR "prevention and control"[All Fields] OR "prevention"[All Fields]))) AND ("1970/01/01"[PDAT] : "2014/01/01"[PDAT])

Flow diagram illustrating the literature selection process

4 citations identified through database searching

0 citations identified trough other sources

4 citations screened

4 full-text articles assessed for eligibility

2 studies included in qualitative synthesis

0 citations excluded

2 full-text articles excluded: Observational study with serious statistical bias of the multivariate model; one letter to the editor

| **Table S1** |  |  |  |
| --- | --- | --- | --- |
| RCT 1 |  | Level of evidence | Very low evidence |
| Year | 2005 | First Author | Acquarolo |
| Journal | ICM |  |  |
| Sample | Ventilated patients, cerebral haemorrhage, GCS < 9 | | |
| Treatment | Ampicillin-sulbactam 3-day course | |  |
| Control | Placebo |  |  |
| Outcome: Desirable effect | Early VAP | |  |
|  |  | Outcome: Desirable effect | |
|  | n° pts | n | % |
| Control | 19 | 4 | 21.1 |
| Treatment | 19 | 11 | 57.9 |
| Total | 38 | 15 | 39.5 |
| Centres | Single Centre | |  |
|  |  | NNTB 3 (95%-CI NNTB 2 to NNTB 17) | |
|  |  | GRADE CRITERIA |  |
| Downgrading | | Allocation concealment | Yes |
|  |  | Intention to treat principle observed | Yes |
|  |  | Blinding | No |
|  |  | Completement of follow-up | Yes |
|  |  | Early stopping | Yes |
|  |  | Selective outcome reporting | Not available |
|  |  | **Bias** | **Very serious** |
|  |  | **Indirectness** | **No** |
|  |  | **Imprecision** | **Very serious** |
|  |  | **Other** | **Very serious** |
|  |  | **Publication bias** | **No** |
|  |  | **Inconsistency with other studies** | **Not assessable** |
| Up-grading | | **Size of effect** | **Large** |
|  |  | **Residual confounding** | **Not applicable** |
|  |  | **Dose /response** | **Not applicable** |
|  |  | DETAILS |  |
| Downgrading | | Blinding: Absence of blinding relevant when the diagnosis is influenced by subjectivity (possible confusion with tracheo-bronchitis); Early stopping: Trial prematurely interrupted on the basis of early-VAP reduction (surrogate/weak outcome) while no difference in mortality was detected; Imprecision: Wide range for both relative-risk and absolute percentages confidence intervals; Other: Very low number of patients (high probability of unbalances in important covariates), single centre trial, resistant bacteria selection not adequately investigated; Inconsistency with other studies: Confidence intervals were excessively wide to assess inconsistency reliably.  The study was downgraded. | |
| Up-grading | | Size of effect: Although the effect is large, there is a high degree of uncertainty that includes negligible effect.  No upgrading was performed. | |

| **Table S1** (continued from the previous page) | | | |
| --- | --- | --- | --- |
| RCT 2 |  | Level of evidence | Very low evidence |
| Year | 1997 | First Author | Kirton |
| Journal | AJRCCM |  |  |
| Sample | Ventilated patients, cerebral haemorrhage, GCS < 13 | | |
| Treatment | 2-dose cefuroxime | |  |
| Control | Placebo |  |  |
| Outcome: Desirable effect | Early VAP | |  |
|  |  | Outcome: Desirable effect | |
|  | n° pts | n | % |
| Control | 50 | 8 | 16.0 |
| Treatment | 50 | 18 | 36.0 |
| Total | 100 | 26 | 26.0 |
| Centres | Single Centre | |  |
|  |  | NNTB 5 (95%-CI NNTB 3 to NNTB 36) | |
|  |  | GRADE CRITERIA |  |
| Downgrading | | Allocation concealment | Not reported |
|  |  | Intention to treat principle observed | Yes |
|  |  | Blinding | No |
|  |  | Completement of follow-up | Yes |
|  |  | Early stopping | No |
|  |  | Selective outcome reporting | Not available |
|  |  | **Bias** | **Serious** |
|  |  | **Indirectness** | **Serious** |
|  |  | **Imprecision** | **Very serious** |
|  |  | **Other** | **Very serious** |
|  |  | **Publication bias** | **No** |
|  |  | **Inconsistency with other studies** | **Not assessable** |
| Up-grading | | **Size of effect** | **Large** |
|  |  | **Residual confounding** | **Not applicable** |
|  |  | **Dose /response** | **Not applicable** |
|  |  | DETAILS |  |
| Downgrading | | Blinding: Absence of blinding relevant when the diagnosis is influenced by subjectivity (possible confusion with tracheo-bronchitis); Imprecision: Wide range for both relative-risk and absolute percentages confidence intervals. Other: low number of patients (high probability of unbalances in important covariates), single centre trial, antibiotic-resistant bacteria selection not adequately investigated; Inconsistency with other studies: Confidence intervals were excessively wide to assess inconsistency reliably.  The study was downgraded. | |
| Up-grading | | Size of effect: Although the effect is large, there is a high degree of uncertainty that includes negligible effect.  No upgrading was performed. | |

| **Abbreviations used in tables and figures in the Supplementary Information section** | |
| --- | --- |
| AAC | *Antimicrobial Agents and Chemotherapy* |
| AJ Surg | *American Journal of Surgery* |
| AJRCCM | *American Journal of Respiratory and Critical Care Medicine* |
| AOS | *Acta Orthopedica Scandinavica* |
| ICM | *Intensive Care Medicine* |
| J.NeuroS | *Journal of Neurosurgery* |
| JOT | *Journal of Orthopedic Trauma* |
| JT | *Journal of Trauma* |
| SurgNeur | *Surgical Neurology* |
| RR | *Relative Risk* |
| NNTB | *Number needed to treat for benefit* |
| NNTH | *Number needed to treat for harm* |
| RCT | *Randomized controlled trial* |
| GCS | *Glasgow Coma Scale* |
| Pts | *Patients* |
