# Supplementary material for: Systematic Review of the Literature and Evidence-Based Recommendations for Antibiotic Prophylaxis in Trauma: Results from an Italian Consensus of Experts
Source: PLoS One. 2014 Nov 20;9(11):e113676. doi: 10.1371/journal.pone.0113676 (PMC4239082; doi:10.1371/journal.pone.0113676)
Supplement: File S2 — MEDLINE database search, flow diagram illustrating the literature selection process, and Table S2 illustrating evidence assessment for the second query. (DOCX) [file pone.0113676.s006.docx]

**File S2:** MEDLINE database search, flow diagram illustrating the literature selection process, and evidence assessment for the second query in **table S2**.

**Question 2**: *is antibiotic prophylaxis effective in patients with basilar skull fractures from non-penetrating head trauma to decrease the occurrence of meningitis?*

**MEDLINE database search:** Clinical trials and observational studies, published since 1970 and written in English, comparing adult patients receiving antibiotic prophylaxis and control groups not receiving any antibacterial drug were selected. Studies on children were excluded.

**PubMed search details**

(((("antibiotic prophylaxis"[MeSH Terms] OR ("antibiotic"[All Fields] AND "prophylaxis"[All Fields]) OR "antibiotic prophylaxis"[All Fields]) OR (("anti-bacterial agents"[Pharmacological Action] OR "anti-bacterial agents"[MeSH Terms] OR ("anti-bacterial"[All Fields] AND "agents"[All Fields]) OR "anti-bacterial agents"[All Fields] OR "antibacterial"[All Fields]) AND ("prevention and control"[Subheading] OR ("prevention"[All Fields] AND "control"[All Fields]) OR "prevention and control"[All Fields] OR "prophylaxis"[All Fields]))) AND (("skull fractures"[MeSH Terms] OR ("skull"[All Fields] AND "fractures"[All Fields]) OR "skull fractures"[All Fields]) OR rhinorrhea[All Fields] OR otorrhea[All Fields] OR ("pneumocephalus"[MeSH Terms] OR "pneumocephalus"[All Fields]))) AND (("infection"[MeSH Terms] OR "infection"[All Fields] OR "communicable diseases"[MeSH Terms] OR ("communicable"[All Fields] AND "diseases"[All Fields]) OR "communicable diseases"[All Fields]) OR ("meningitis"[MeSH Terms] OR "meningitis"[All Fields]))) AND ("1970/01/01"[PDAT] : "2014/01/01"[PDAT])

Flow diagram illustrating the literature selection process

7 full-text articles excluded: 4 observational studies not performing adjusted comparisons of outcomes; 3 meta-analyses including studies excessively heterogeneous in terms of design

136 citations excluded

3 studies included in qualitative synthesis

10 full-text articles assessed for eligibility

146 citations screened

0 citations identified trough other sources

146 citations identified through database searching

| **Table S2** |  |  |  |
| --- | --- | --- | --- |
| RCT 1 |  | Level of evidence | No evidence |
| Year | 1992 | First Author | Demetriades |
| Journal | Injury |  |  |
| Sample | Basilar skull fractures | |  |
| Treatment | 3-day course ceftriaxone or ampicillin/sulphadiazine | | |
| Control | No placebo |  |  |
| Outcome: Desirable effect | Meningitis reduction | |  |
|  |  | Outcome: Desirable effect | |
|  | n° pts | n | % |
| Treatment | 25 | 0 | 0.0 |
| Control | 12 | 1 | 8.3 |
| Total | 37 | 1 | 2.7 |
| Centres | Single Centre | |  |
|  |  | NNTB 12 (95%-CI NNTB 3 to ∞ to NNTH 15) | |
|  |  | GRADE CRITERIA |  |
| Downgrading | | Allocation concealment | Not reported |
|  |  | Intention to treat principle observed | Not reported |
|  |  | Blinding | No |
|  |  | Completement of follow-up | No |
|  |  | Early stopping | No |
|  |  | Selective outcome reporting | Not available |
|  |  | **Bias** | **Very serious** |
|  |  | **Indirectness** | **No** |
|  |  | **Imprecision** | **Very serious** |
|  |  | **Other** | **Very serious** |
|  |  | **Publication bias** | **No** |
|  |  | **Inconsistency with other trials** | **Not assessable** |
| Up-grading | | **Size of effect** | **Not relevant** |
|  |  | **Residual confounding** | **Not assessable** |
|  |  | **Dose /response** | **Not applicable** |
|  |  | DETAILS |  |
| Downgrading | | Completement of follow-up: 39 withdrawals, 31 lost to follow-up; Other: Subgroup analysis of 196 patients originally included in the study; no placebo; very small sample size that compromises the distribution of important variables between study arms and the power to detect clinically meaningful differences; Inconsistency with other trials: The confidence intervals of absolute proportion differences are wide (difference not assessable).  The study was downgraded. | |
| Up-grading | | No upgrading was performed. | |

| **Table S2** (continued from the previous page) | | | |
| --- | --- | --- | --- |
| RCT 2 |  | Level of evidence | No evidence |
| Year | 2004 | First Author | Eftekhar |
| Journal | J.NeuroS |  |  |
| Sample | Acute traumatic pneumocephalus verified by CT scan | | |
| Treatment | 5-day course ceftriaxone | |  |
| Control | No placebo |  |  |
| Outcome: Desirable effect | Meningitis reduction | |  |
|  |  | Outcome: Desirable effect | |
|  | n° pts | n | % |
| Treatment | 53 | 10 | 18.9 |
| Control | 56 | 12 | 21.4 |
| Total | 109 | 22 | 20.2 |
| Centres | Single Centre | |  |
|  |  | NNTB 39 (95%-CI NNTB 6 to ∞ to NNTH 8) | |
|  |  | GRADE CRITERIA |  |
| Downgrading | | Allocation concealment | Not reported |
|  |  | Intention to treat principle observed | Not reported |
|  |  | Blinding | No |
|  |  | Completement of follow-up | Yes |
|  |  | Early stopping | No |
|  |  | Selective outcome reporting | Not available |
|  |  | **Bias** | **Very serious** |
|  |  | **Indirectness** | **No** |
|  |  | **Imprecision** | **Serious** |
|  |  | **Other** | **No** |
|  |  | **Publication bias** | **No** |
|  |  | **Inconsistency with other trials** | **Not assessable** |
| Up-grading | | **Size of effect** | **Not relevant** |
|  |  | **Residual confounding** | **Not assessable** |
|  |  | **Dose /response** | **Not applicable** |
|  |  | DETAILS |  |
| Downgrading | | Other: Small sample size that may compromise the distribution of important variables between study arms, there is a very high rate of meningitis compared to the other studies, basilar skull fractures were not specified, 5-day antibiotic course, no monitoring of resistant bacteria; no placebo. Inconsistency with other trials: The imprecision was high (confidence intervals of absolute proportion differences wide) and thus the difference not assessable.  The study was downgraded. | |
| Up-grading | | No upgrading was performed. | |

| **Table S2** (continued from the previous page) | | | |
| --- | --- | --- | --- |
| RCT 3 |  | Level of evidence | No evidence |
| Year | 1976 | First Author | Klastersky |
| Journal | SurgNeur |  |  |
| Sample | Traumatic rhinorrhoea or otorrhoea | |  |
| Treatment | Average 7.7 days course penicillin | |  |
| Control | Placebo |  |  |
| Outcome: Desirable effect | Meningitis reduction | |  |
|  |  | Outcome: Desirable effect | |
|  | n° pts | n | % |
| Treatment | 26 | 0 | 0.0 |
| Control | 26 | 1 | 3.8 |
| Total | 52 | 1 | 1.9 |
| Centres | Single Centre | |  |
|  |  | NNTB 26 (95%-CI NNTB 5 to ∞ to NNTH 11) | |
|  |  | GRADE CRITERIA |  |
| Downgrading | | Allocation concealment | Not reported |
|  |  | Intention to treat principle observed | Not reported |
|  |  | Blinding | Yes |
|  |  | Completement of follow-up | Yes |
|  |  | Early stopping | No |
|  |  | Selective outcome reporting | Not available |
|  |  | **Bias** | **Serious** |
|  |  | **Indirectness** | **No** |
|  |  | **Imprecision** | **Serious** |
|  |  | **Other** | **No** |
|  |  | **Publication bias** | **No** |
|  |  | **Inconsistency with other trials** | **Not assessable** |
| Up-grading | | **Size of effect** | **Not relevant** |
|  |  | **Residual confounding** | **Not assessable** |
|  |  | **Dose /response** | **Not applicable** |
|  |  | DETAILS |  |
| Downgrading | | Bias: Very small sample size that may compromise the distribution of important variables between study arms and the power to detect clinically meaningful differences, average 7.7 day antibiotic administration and no resistant bacteria monitoring; Inconsistency with other trials: The confidence intervals of absolute proportion differences are wide (difference not assessable).  The study was downgraded. | |
| Up-grading | | No upgrading was performed. | |

| **Abbreviations used in tables and figures in the Supplementary Information section** | |
| --- | --- |
| AAC | *Antimicrobial Agents and Chemotherapy* |
| AJ Surg | *American Journal of Surgery* |
| AJRCCM | *American Journal of Respiratory and Critical Care Medicine* |
| AOS | *Acta Orthopedica Scandinavica* |
| ICM | *Intensive Care Medicine* |
| J.NeuroS | *Journal of Neurosurgery* |
| JOT | *Journal of Orthopedic Trauma* |
| JT | *Journal of Trauma* |
| SurgNeur | *Surgical Neurology* |
| RR | *Relative Risk* |
| NNTB | *Number needed to treat for benefit* |
| NNTH | *Number needed to treat for harm* |
| RCT | *Randomized controlled trial* |
| GCS | *Glasgow Coma Scale* |
| Pts | *Patients* |
