# Supplementary material for: Systematic Review of the Literature and Evidence-Based Recommendations for Antibiotic Prophylaxis in Trauma: Results from an Italian Consensus of Experts
Source: PLoS One. 2014 Nov 20;9(11):e113676. doi: 10.1371/journal.pone.0113676 (PMC4239082; doi:10.1371/journal.pone.0113676)
Supplement: File S3 — MEDLINE database search, flow diagram illustrating the literature selection process, and Table S3 illustrating evidence assessment for the third query. (DOCX) [file pone.0113676.s007.docx]

**File S3:** MEDLINE database search, flow diagram illustrating the literature selection process, and evidence assessment for the third query in **table S3**.

**Question 3**: *is antibiotic prophylaxis beneficial for the reduction of wound infection rate in patients with long-bone open fractures?*

**MEDLINE database search:** clinical trials and observational studies, published since 1970 and written in English, comparing patients receiving antibiotic prophylaxis and control groups not receiving any antibacterial drug were selected. Pediatric and animal studies were excluded. Reviews, and letters to the editor were not considered.

**PubMed search details**

(((("open fractures"[All Fields] OR "extremity injury"[All Fields]) AND (("anti-bacterial agents"[Pharmacological Action] OR "anti-bacterial agents"[MeSH Terms] OR ("anti-bacterial"[All Fields] AND "agents"[All Fields]) OR "anti-bacterial agents"[All Fields] OR "antibiotic"[All Fields]) OR ("anti-infective agents"[Pharmacological Action] OR "anti-infective agents"[MeSH Terms] OR ("anti-infective"[All Fields] AND "agents"[All Fields]) OR "anti-infective agents"[All Fields] OR "antimicrobial"[All Fields]))) AND ("prevention and control"[Subheading] OR ("prevention"[All Fields] AND "control"[All Fields]) OR "prevention and control"[All Fields] OR "prophylaxis"[All Fields])) AND english[Language]) AND ("1970/01/01"[PDAT] : "2014/01/01"[PDAT])

Flow diagram illustrating the literature selection process

3 full text articles excluded: One low quality trial; one observational study reporting only crude outcomes; one meta-analysis including studies excessively heterogeneous in terms of design.

169 citations excluded

2 studies included in qualitative synthesis

5 full-text articles assessed for eligibility

174 citations screened

4 citations identified trough other sources

170 citations identified through database searching

| **Table S3** |  |  |  |
| --- | --- | --- | --- |
| RCT 1 |  | Level of evidence | No evidence |
| Year | 1982 | First Author | Bergman |
| Journal | AOS |  |  |
| Sample | Long-bone open fractures | |  |
| Treatment | 48-hour course dicoxacillin or penicillin | |  |
| Control | Placebo |  |  |
| Outcome: Desirable effect | Wound infection (not specifically osteomyelitis) | |  |
|  |  | Outcome: Desirable effect | |
|  | n° pts | n | % |
| Treatment | 60 | 4 | 6.7 |
| Control | 30 | 6 | 20.0 |
| Total | 90 | 10 | 11.1 |
| Centres | Single Centre | |  |
|  |  | NNTB 8 (95%-CI NNTB 3 to ∞ to NNTH 151) | |
|  |  | GRADE CRITERIA |  |
| Downgrading | | Allocation concealment | Yes |
|  |  | Intention to treat principle observed | No |
|  |  | Blinding | Yes |
|  |  | Completement of follow-up | Yes |
|  |  | Early stopping | No |
|  |  | Selective outcome reporting | Not available |
|  |  | **Bias** | **Serious** |
|  |  | **Indirectness** | **No** |
|  |  | **Imprecision** | **Serious** |
|  |  | **Other** | **Very serious** |
|  |  | **Publication bias** | **No** |
|  |  | **Inconsistency with other trials** | **Not assessable** |
| Up-grading | | **Size of effect** | **Large** |
|  |  | **Residual confounding** | **Not assessable** |
|  |  | **Dose /response** | **Not applicable** |
|  |  | DETAILS |  |
| Downgrading | | Intention to treat principle: five patients were excluded from the study because of flocculation of antibiotic; Imprecision: Confidence intervals for absolute differences were wide; Other: Subgroup analysis of a larger trial including closed fractures, low number of patients (high probability of unbalances in important covariates and low power), single centre trial, antibiotic-resistant bacteria selection not adequately investigated; Inconsistency with other trials: Confidence intervals were excessively wide to assess inconsistency reliably.  The study was downgraded. | |
| Up-grading | | Size of effect: Although the effect is large, there is a high degree of uncertainty that includes negligible effect.  No upgrading performed. | |

| **Table S3** (continued from the previous page) | | | |
| --- | --- | --- | --- |
| RCT 2 |  | Level of evidence | Low evidence |
| Year | 1987 | First Author | Braun |
| Journal | JOT |  |  |
| Sample | Long-bone open fractures | |  |
| Treatment | 10-day course cloxacillin | |  |
| Control | Placebo |  |  |
| Outcome: Desirable effect | Wound infection (not specifically osteomyelitis) | |  |
|  |  | Outcome: Desirable effect | |
|  | n° pts | n | % |
| Treatment | 43 | 2 | 4.7 |
| Control | 44 | 12 | 27.3 |
| Total | 87 | 14 | 16.1 |
| Centres | Single Centre | |  |
|  |  | NNTB 4 (95%-CI NNTB 3 to NNTB 14) | |
|  |  | GRADE CRITERIA |  |
| Downgrading | | Allocation concealment | Not reported |
|  |  | Intention to treat principle observed | Yes |
|  |  | Blinding | Yes |
|  |  | Completement of follow-up | Yes |
|  |  | Early stopping | No |
|  |  | Selective outcome reporting | Not available |
|  |  | **Bias** | **Serious** |
|  |  | **Indirectness** | **No** |
|  |  | **Imprecision** | **Serious** |
|  |  | **Other** | **Serious** |
|  |  | **Publication bias** | **No** |
|  |  | **Inconsistency with other trials** | **Not assessable** |
| Up-grading | | **Size of effect** | **Very large** |
|  |  | **Residual confounding** | **Not assessable** |
|  |  | **Dose /response** | **Not applicable** |
|  |  | DETAILS |  |
| Downgrading | | Imprecision: Confidence intervals for absolute differences are wide; Other: Method for random sequence generation not reported, very low number of patients (high probability of unbalances in important covariates), single centre trial, antibiotic-resistant bacteria selection not adequately investigated; infections occurred within six weeks from trauma were considered a very long period to be in relation with initial antibiotic prophylaxis; Inconsistency with other trials: Confidence intervals were excessively wide to assess inconsistency reliably.  The study was downgraded. | |
| Up-grading | | Size of effect: Although the effect is very large, there is a high degree of uncertainty that includes negligible effect.  No upgrading was performed. | |

| **Abbreviations used in tables and figures in the Supplementary Information section** | |
| --- | --- |
| AAC | *Antimicrobial Agents and Chemotherapy* |
| AJ Surg | *American Journal of Surgery* |
| AJRCCM | *American Journal of Respiratory and Critical Care Medicine* |
| AOS | *Acta Orthopedica Scandinavica* |
| ICM | *Intensive Care Medicine* |
| J.NeuroS | *Journal of Neurosurgery* |
| JOT | *Journal of Orthopedic Trauma* |
| JT | *Journal of Trauma* |
| SurgNeur | *Surgical Neurology* |
| RR | *Relative Risk* |
| NNTB | *Number needed to treat for benefit* |
| NNTH | *Number needed to treat for harm* |
| RCT | *Randomized controlled trial* |
| GCS | *Glasgow Coma Scale* |
| Pts | *Patients* |
