# Supplementary material for: Systematic Review of the Literature and Evidence-Based Recommendations for Antibiotic Prophylaxis in Trauma: Results from an Italian Consensus of Experts
Source: PLoS One. 2014 Nov 20;9(11):e113676. doi: 10.1371/journal.pone.0113676 (PMC4239082; doi:10.1371/journal.pone.0113676)
Supplement: File S4 — MEDLINE database search, flow diagram illustrating the literature selection process, and Table S4 illustrating evidence assessment for the fourth query. (DOCX) [file pone.0113676.s008.docx]

**File S4:** MEDLINE database search, flow diagram illustrating the literature selection process, and evidence assessment for the fourth query in **table S4**.

**Question 4**: *is antibiotic prophylaxis indicated to reduce the risk of deep surgical site infections in patients with abdominal trauma and enteric abdominal contamination submitted to emergent surgery****?***

**MEDLINE database search:** clinical trials and observational studies, published since 1970 and written in English, comparing patients receiving antibiotic prophylaxis and control groups not receiving any antibacterial drug were selected. Reviews, case reports, pediatric studies were excluded.

**PubMed search details**

(((("abdominal injuries"[MeSH Terms] OR ("abdominal"[All Fields] AND "injuries"[All Fields]) OR "abdominal injuries"[All Fields] OR ("abdominal"[All Fields] AND "trauma"[All Fields]) OR "abdominal trauma"[All Fields]) OR ("abdominal injuries"[MeSH Terms] OR ("abdominal"[All Fields] AND "injuries"[All Fields]) OR "abdominal injuries"[All Fields] OR ("abdominal"[All Fields] AND "injury"[All Fields]) OR "abdominal injury"[All Fields]) OR (blunt[All Fields] AND ("abdominal injuries"[MeSH Terms] OR ("abdominal"[All Fields] AND "injuries"[All Fields]) OR "abdominal injuries"[All Fields] OR ("abdominal"[All Fields] AND "trauma"[All Fields]) OR "abdominal trauma"[All Fields])) OR (penetrating[All Fields] AND ("abdominal injuries"[MeSH Terms] OR ("abdominal"[All Fields] AND "injuries"[All Fields]) OR "abdominal injuries"[All Fields] OR ("abdominal"[All Fields] AND "trauma"[All Fields]) OR "abdominal trauma"[All Fields]))) AND (("surgical wound infection"[MeSH Terms] OR ("surgical"[All Fields] AND "wound"[All Fields] AND "infection"[All Fields]) OR "surgical wound infection"[All Fields] OR ("surgical"[All Fields] AND "site"[All Fields] AND "infection"[All Fields]) OR "surgical site infection"[All Fields]) OR ("infection"[MeSH Terms] OR "infection"[All Fields] OR "communicable diseases"[MeSH Terms] OR ("communicable"[All Fields] AND "diseases"[All Fields]) OR "communicable diseases"[All Fields]) OR ("peritonitis"[MeSH Terms] OR "peritonitis"[All Fields]) OR (hollow[All Fields] AND ("viscera"[MeSH Terms] OR "viscera"[All Fields] OR "viscus"[All Fields]) AND perforation[All Fields]) OR ("intestinal perforation"[MeSH Terms] OR ("intestinal"[All Fields] AND "perforation"[All Fields]) OR "intestinal perforation"[All Fields] OR ("bowel"[All Fields] AND "perforation"[All Fields]) OR "bowel perforation"[All Fields]))) AND (("antibiotic prophylaxis"[MeSH Terms] OR ("antibiotic"[All Fields] AND "prophylaxis"[All Fields]) OR "antibiotic prophylaxis"[All Fields]) OR (("anti-bacterial agents"[Pharmacological Action] OR "anti-bacterial agents"[MeSH Terms] OR ("anti-bacterial"[All Fields] AND "agents"[All Fields]) OR "anti-bacterial agents"[All Fields] OR "antibacterial"[All Fields]) AND ("prevention and control"[Subheading] OR ("prevention"[All Fields] AND "control"[All Fields]) OR "prevention and control"[All Fields] OR "prophylaxis"[All Fields])) OR (anti[All Fields] AND microbial[All Fields] AND ("prevention and control"[Subheading] OR ("prevention"[All Fields] AND "control"[All Fields]) OR "prevention and control"[All Fields] OR "prophylaxis"[All Fields])) OR ("prevention and control"[Subheading] OR ("prevention"[All Fields] AND "control"[All Fields]) OR "prevention and control"[All Fields] OR "prevention"[All Fields]))) AND ("1970/01/01"[PDAT] : "2014/01/01"[PDAT])

Flow diagram illustrating the literature selection process

1 full-text article excluded: RCT not reporting the deep surgical-site infection rate

501 citations excluded

3 studies included in qualitative synthesis

4 full-text articles assessed for eligibility

505 citations screened

1 citation identified trough other sources

504 citations identified through database searching

| **Table S4** |  |  |  |
| --- | --- | --- | --- |
| RCT 1 |  | Level of evidence | Very low evidence |
| Year | 1992 | First Author | Fabian |
| Journal | Surgery |  |  |
| Sample | Penetrating abdominal trauma | |  |
| Treatment | 24-hour cefoxitin or cefotetan | |  |
| Control | 5-day cefoxitin or cefotetan | |  |
| Outcome: Desirable effect | Deep surgical site infections | |  |
|  |  | Outcome: Desirable effect | |
|  | n° pts | n | % |
| Treatment | 265 | 21 | 7.9 |
| Control | 250 | 25 | 10.0 |
| Total | 515 | 46 | 8.9 |
| Centres | Single centre | |  |
|  |  | NNTB 48 (95%-CI NNTB 14 to ∞ to NNTH 34) | |
|  |  | GRADE CRITERIA |  |
| Downgrading | | Allocation concealment | Not reported |
|  |  | Intention to treat principle observed | Not reported |
|  |  | Blinding | Yes |
|  |  | Completement of follow-up | Yes |
|  |  | Early stopping | No |
|  |  | Selective outcome reporting | Not available |
|  |  | **Bias** | **Serious** |
|  |  | **Indirectness** | **No** |
|  |  | **Imprecision** | **No** |
|  |  | **Other** | **Very serious** |
|  |  | **Publication bias** | **No** |
|  |  | **Inconsistency with other trials** | **No** |
| Up-grading | | **Size of effect** | **Not relevant** |
|  |  | **Residual confounding** | **Not assessable** |
|  |  | **Dose /response** | **Not applicable** |
|  |  | DETAILS |  |
| Downgrading | | Other: Underpowered to detect clinically meaningful differences. Patients with perforation were the minority in the two study arms, the distribution of deep surgical site infections among them was not reported and no subgroup analysis was performed.  The study was downgraded. | |
| Up-grading | | No upgrading was performed. | |

| **Table S4** (continued from the previous page) | | | | |
| --- | --- | --- | --- | --- |
| RCT 2 |  | Level of evidence | | Very low evidence |
| Year | 2000 | First Author | | Kirton |
| Journal | JT |  | |  |
| Sample | Penetrating abdominal trauma | | |  |
| Treatment | 24-hour ampicillin-sulbactam | | |  |
| Control | 5-day ampicillin-sulbactam | | |  |
| Outcome: Desirable effect | Deep surgical site infections | | |  |
|  |  | | Outcome: Desirable effect | |
|  | n° pts | | n | % |
| Treatment | 158 | | 13 | 8.2 |
| Control | 159 | | 16 | 10.1 |
| Total | 317 | | 29 | 9.1 |
| Centres | 4 Centres | |  |  |
| Power | 0.082 | | NNTB 54 (95%-CI NNTB 12 to ∞ to NNTH 21) | |
|  |  | | GRADE CRITERIA |  |
| Downgrading | | | Allocation concealment | Not reported |
|  |  |  | Intention to treat principle observed | Not reported |
|  |  |  | Blinding | Yes |
|  |  |  | Completement of follow-up | Yes |
|  |  |  | Early stopping | No |
|  |  |  | Selective outcome reporting | Not available |
|  |  |  | **Bias** | **Serious** |
|  |  |  | **Indirectness** | **No** |
|  |  |  | **Imprecision** | **No** |
|  |  |  | **Other** | **Very serious** |
|  |  |  | **Publication bias** | **No** |
|  |  |  | **Inconsistency with other trials** | **No** |
| Up-grading | | | **Size of effect** | **Not relevant** |
|  |  |  | **Residual confounding** | **Not assessable** |
|  |  |  | **Dose /response** | **Not applicable** |
|  |  | | DETAILS |  |
| Downgrading | | | Other: Patients with perforation were at least 50% in the two study arms, the distribution of deep surgical site infections among them was not reported and no subgroup analysis was performed. Moreover, those with perforation were 54 (34%) in the short-prophylaxis study arm and 72 (45%) in the 5-day administration group, an 11% statistically significant difference (95%-CI 0.3 to 21.5); this unbalance may have affected the result; Underpowered to detect clinically meaningful differences.  The study was downgraded. | |
| Up-grading | | | No upgrading was performed. | |

| RCT 3 |  | Level of evidence | Very low evidence |
| --- | --- | --- | --- |
| Year | 1999 | First Author | Bozorgzedeh |
| Journal | AJ Surg |  |  |
| Sample | Penetrating abdominal trauma | |  |
| Treatment | 24-hour cefoxitin | |  |
| Control | 5-day cefoxitin | |  |
| Outcome: Desirable effect | Deep surgical site infections | |  |
|  |  | Outcome: Desirable effect | |
|  | n° pts | n | % |
| Treatment | 148 | 9 | 6.1 |
| Control | 152 | 9 | 5.9 |
| Total | 300 | 18 | 6 |
| Centres | 2 Centres | |  |
| Power | 0.029 | NNTH 625 (95%-CI NNTB 18 to ∞ to NNTH 17) | |
|  |  | GRADE CRITERIA |  |
| Downgrading | | Allocation concealment | Not reported |
|  |  | Intention to treat principle observed | Not reported |
|  |  | Blinding | No |
|  |  | Completement of follow-up | Yes |
|  |  | Early stopping | No |
|  |  | Selective outcome reporting | Not available |
|  |  | **Bias** | **Very serious** |
|  |  | **Indirectness** | **No** |
|  |  | **Imprecision** | **No** |
|  |  | **Other** | **Very serious** |
|  |  | **Publication bias** | **No** |
|  |  | **Inconsistency with other trials** | **No** |
| Up-grading | | **Size of effect** | **Not relevant** |
|  |  | **Residual confounding** | **Not assessable** |
|  |  | **Dose /response** | **Not applicable** |
|  |  | DETAILS |  |
| Downgrading | | No placebo was administered after the 24-hour treatment. Other: Patients with perforation were about 50% in the two study arms, and the distribution of deep surgical site infections among them was not reported and no subgroup analysis was performed. Underpowered to detect clinically meaningful differences.  The study was downgraded. | |
| Up-grading | | No upgrading was performed. | |

| **Abbreviations used in tables and figures in the Supplementary Information section** | |
| --- | --- |
| AAC | *Antimicrobial Agents and Chemotherapy* |
| AJ Surg | *American Journal of Surgery* |
| AJRCCM | *American Journal of Respiratory and Critical Care Medicine* |
| AOS | *Acta Orthopedica Scandinavica* |
| ICM | *Intensive Care Medicine* |
| J.NeuroS | *Journal of Neurosurgery* |
| JOT | *Journal of Orthopedic Trauma* |
| JT | *Journal of Trauma* |
| SurgNeur | *Surgical Neurology* |
| RR | *Relative Risk* |
| NNTB | *Number needed to treat for benefit* |
| NNTH | *Number needed to treat for harm* |
| RCT | *Randomized controlled trial* |
| GCS | *Glasgow Coma Scale* |
| Pts | *Patients* |
