# Supplementary material for: Systematic Review of the Literature and Evidence-Based Recommendations for Antibiotic Prophylaxis in Trauma: Results from an Italian Consensus of Experts
Source: PLoS One. 2014 Nov 20;9(11):e113676. doi: 10.1371/journal.pone.0113676 (PMC4239082; doi:10.1371/journal.pone.0113676)
Supplement: File S5 — MEDLINE database search, flow diagram illustrating the literature selection process, and Table S5 illustrating evidence assessment for antibiotic-resistant bacteria (undesirable effect). (DOCX) [file pone.0113676.s009.docx]

**File S5:** Short antibiotic prophylaxis and resistant bacteria selection (undesirable effect)

The question addressing the unfavourable effects of antibiotic prophylaxis was: *Does short antibiotic prophylaxis determine resistant bacteria selection?*

*Medline database search:* clinical trials and observational studies, published since 1970 and written in English, comparing patients receiving antibiotic prophylaxis and control groups not receiving any antibacterial drug were selected. The research was limited to studies concerning humans. Pediatric studies were excluded. Letters to the editor were not considered.

**PubMed search details**

((((("bacterial colonization"[All Fields] OR "colonization"[All Fields]) AND (("antibiotic prophylaxis"[MeSH Terms] OR ("antibiotic"[All Fields] AND "prophylaxis"[All Fields]) OR "antibiotic prophylaxis"[All Fields]) OR ("anti-bacterial agents"[Pharmacological Action] OR "anti-bacterial agents"[MeSH Terms] OR ("anti-bacterial"[All Fields] AND "agents"[All Fields]) OR "anti-bacterial agents"[All Fields] OR ("anti"[All Fields] AND "bacterial"[All Fields] AND "agents"[All Fields]) OR "anti bacterial agents"[All Fields]))) AND "resistant bacteria"[All Fields]) OR (("Anti-Bacterial Agents/administration and dosage"[MeSH Terms] AND "Drug Resistance, Microbial"[MAJR]) AND "Antibiotic Prophylaxis"[MAJR])) OR ("Postoperative Complications/epidemiology"[MAJR] AND "Premedication"[MAJR])) AND ("1970/01/01"[PDAT] : "2014/01/01"[PDAT])

Flow diagram illustrating the literature selection process

0 full-text articles excluded

271 citations excluded

3 studies included in qualitative synthesis

3 full-text articles assessed for eligibility

274 citations screened

0 citations identified trough other sources

274 citations identified through database searching

| **Table S5** |  |  |  |
| --- | --- | --- | --- |
| Observational: before/after |  | Level of evidence | Low evidence |
| Year | 2009 | First Author | Tacconelli |
| Journal | AAC |  |  |
| Sample | Patients exposed to antibiotics without previous antibiotic-resistant bacteria colonization | | |
| Treatment | Mainly cephalosporins, quinolones, macrolides | | |
| Control | No |  |  |
| Outcome | Acquisition of antibiotic-resistant bacteria | |  |
|  | n° pts | n | % |
| Control |  |  |  |
| Treatment |  |  |  |
| Total | 864 |  |  |
| Centres | 5 |  |  |
|  |  | GRADE CRITERIA |  |
| Downgrading | | Allocation concealment | 0 |
|  |  | Intention to treat principle observed | 0 |
|  |  | Blinding | 0 |
|  |  | Completement of follow-up | 0 |
|  |  | Early stopping | 0 |
|  |  | **Bias** | **No** |
|  |  | **Indirectness** | **No** |
|  |  | **Imprecision** | **No** |
|  |  | **Other** | **No** |
|  |  | **Publication bias** | **No** |
|  |  | **Inconsistency with other studies** | **Not assessable** |
| Up-grading | | **Size of effect** | **Not assessable** |
|  |  | **Residual confounding** | **Does not indicate upgrading** |
|  |  | **Dose /response** | **Not applicable** |
|  |  | DETAILS |  |
| Downgrading | | Bias: It is unlikely that temporal trends and other factors besides antibiotic exposure could affect 48-hour colonization. The absence of control group is not considered crucial for this endpoint.  No downgrading was performed. | |
| Up-grading | | Size of effect: Limited rate increase of antibiotic resistance.  No upgrading was performed. | |

| **Table S5** (continued from the previous page) | | | |
| --- | --- | --- | --- |
| Observational: before/after |  | Level of evidence | Very low evidence |
| Year | 1988 | First Author | Kernodle |
| Journal | AAC |  |  |
| Sample | Patients exposed to antibiotics without or with limited previous antibiotic-resistant bacteria colonization | | |
| Treatment | Cephalosporins | |  |
| Control | No |  |  |
| Outcome | Acquisition of antibiotic-resistant bacteria | |  |
|  | n° pts | n | % |
| Control |  |  |  |
| Treatment |  |  |  |
| Total | 39 |  |  |
| Centres | 1 |  |  |
|  |  | GRADE CRITERIA |  |
| Downgrading | | Allocation concealment | 0 |
|  |  | Intention to treat principle observed | 0 |
|  |  | Blinding | 0 |
|  |  | Completement of follow-up | 0 |
|  |  | Early stopping | 0 |
|  |  | **Bias** | **Very serious** |
|  |  | **Indirectness** | **No** |
|  |  | **Imprecision** | **No** |
|  |  | **Other** | **No** |
|  |  | **Publication bias** | **No** |
|  |  | **Inconsistency with other studies** | **Not assessable** |
| Up-grading | | **Size of effect** | **Not assessable** |
|  |  | **Residual confounding** | **Does not indicate upgrading** |
|  |  | **Dose /response** | **Not applicable** |
|  |  | DETAILS |  |
| Downgrading | | Bias: Multiple samples taken from each patient, quantitative assessment impossible. No control group, it is unlikely however that temporal trends and other factors besides antibiotic exposure could affect 48-hour colonization. Very small sample size.  The study was downgraded. | |
| Up-grading | | No upgrading was performed. | |

| **Table S5** (continued from the previous page) | | | |
| --- | --- | --- | --- |
| Observational:  logistic regression | | Level of evidence | Very low evidence |
| Year | 2000 | First Author | Harbarth |
| Journal | Circulation | |  |
| Sample | Patients exposed to antibiotics without or with limited previous antibiotic-resistant bacteria colonization | | |
| Treatment | Cephalosporins | |  |
| Control | No |  |  |
| Outcome | Acquisition of antibiotic-resistant bacteria | |  |
|  |  |  | |
|  | n° pts | n | % |
| Control |  |  |  |
| Treatment |  |  |  |
| Total | 1094 |  |  |
| Centres | 1 |  |  |
|  |  | GRADE CRITERIA |  |
| Downgrading | | Allocation concealment | 0 |
|  |  | Intention to treat principle observed | 0 |
|  |  | Blinding | 0 |
|  |  | Completement of follow-up | 0 |
|  |  | Early stopping | 0 |
|  |  | **Bias** | **Very serious** |
|  |  | **Indirectness** | **No** |
|  |  | **Imprecision** | **No** |
|  |  | **Other** | **No** |
|  |  | **Publication bias** | **Not assessable** |
|  |  | **Inconsistency with other studies** | **Not assessable** |
| Up-grading | | **Size of effect** | **Not assessable** |
|  |  | **Residual confounding** | **Does not indicate upgrading** |
|  |  | **Dose /response** | **Not applicable** |
|  |  | DETAILS |  |
| Downgrading | | Bias: Scanty reporting of statistics. Potential selection bias not adjusted for by logistic regression.  The study was downgraded. | |
| Up-grading | | Size of effect: Only ODDS ratios from logistic regression available not indicating large size effect.  No upgrading was performed. | |
