# Supplementary material for: Systematic Review of the Literature and Evidence-Based Recommendations for Antibiotic Prophylaxis in Trauma: Results from an Italian Consensus of Experts
Source: PLoS One. 2014 Nov 20;9(11):e113676. doi: 10.1371/journal.pone.0113676 (PMC4239082; doi:10.1371/journal.pone.0113676)
Supplement: File S6 — Preferred Reporting Items for Systematic Reviews and Meta-Analyses (PRISMA) statement recommendations checklist (Table S6). (DOC) [file pone.0113676.s010.doc]

**File S6:** In this file we report the Checklist of items included in our systematic review according to the Preferred Reporting Items for Systematic Reviews and Meta-Analyses (PRISMA) statement recommendations.

We considered the retrieved studies too heterogeneous particularly in terms of design to be combined in meta-analyses, which hence have not been performed. We also were unable to formally assess the publication risk of bias, because the number of study retrieved was insufficient to gain sufficient power for the test. We instead complied with all the other recommendations. The main limitation of the review was that we searched only the MEDLINE database using the free PubMed provider.

| Table S6 |  |  |  |
| --- | --- | --- | --- |
| Section/topic | # | Checklist item | Reported on page # |
| **TITLE** | | | |
| Title | 1 | Identify the report as a systematic review, meta-analysis, or both. | Page 1 |
| **ABSTRACT** | | | |
| Structured summary | 2 | Provide a structured summary including, as applicable: background; objectives; data sources; study eligibility criteria, participants, and interventions; study appraisal and synthesis methods; results; limitations; conclusions and implications of key findings; systematic review registration number. | Page 2 |
| **INTRODUCTION** | | | |
| Rationale | 3 | Describe the rationale for the review in the context of what is already known. | Page 4, first and third paragraph. |
| Objectives | 4 | Provide an explicit statement of questions being addressed with reference to participants, interventions, comparisons, outcomes, and study design (PICOS). | Page 4, first and third paragraph. Questions formulated in the first paragraph of pages 13, 15, 17, and 20. Supplementary information tables from 1 to 5. |
| **METHODS** | | | |
| Protocol and registration | 5 | Indicate if a review protocol exists, if and where it can be accessed (e.g., Web address), and, if available, provide registration information including registration number. | Page 6, second paragraph. |
| Eligibility criteria | 6 | Specify study characteristics (e.g., PICOS, length of follow-up) and report characteristics (e.g., years considered, language, publication status) used as criteria for eligibility, giving rationale. | Pages 5 and 6 |
| Information sources | 7 | Describe all information sources (e.g., databases with dates of coverage, contact with study authors to identify additional studies) in the search and date last searched. | Pages 5, third, fourth, and fifth paragraphs |
| Search | 8 | Present full electronic search strategy for at least one database, including any limits used, such that it could be repeated. | Supplementary information tables from 1 to 5 |
| Study selection | 9 | State the process for selecting studies (i.e., screening, eligibility, included in systematic review, and, if applicable, included in the meta-analysis). | Pages 5 and 6 |
| Data collection process | 10 | Describe method of data extraction from reports (e.g., piloted forms, independently, in duplicate) and any processes for obtaining and confirming data from investigators. | Page 6, second paragraph. Supplementary information tables from 1 to 5. |
| Data items | 11 | List and define all variables for which data were sought (e.g., PICOS, funding sources) and any assumptions and simplifications made. | Page 6, third paragraph. Supplementary information tables from 1 to 5. |
| Risk of bias in individual studies | 12 | Describe methods used for assessing risk of bias of individual studies (including specification of whether this was done at the study or outcome level), and how this information is to be used in any data synthesis. | Page 6, second paragraph. Page 7, second and third paragraphs. Page 8. Supplementary information tables from 1 to 5. |
| Summary measures | 13 | State the principal summary measures (e.g., risk ratio, difference in means). | Page 6, third paragraph. |
| Synthesis of results | 14 | Describe the methods of handling data and combining results of studies, if done, including measures of consistency (e.g., I^2^) for each meta-analysis. | Data were not combined because of excessive design heterogeneity between studies and the limited number of studies. Page 6, fifth paragraph. |
| Risk of bias across studies | 15 | Specify any assessment of risk of bias that may affect the cumulative evidence (e.g., publication bias, selective reporting within studies). | Not applicable because of the insufficient number of studies needed to perform a formal approach. Page 6, fifth paragraph. |
| Additional analyses | 16 | Describe methods of additional analyses (e.g., sensitivity or subgroup analyses, meta-regression), if done, indicating which were pre-specified. | No additional analyses were performed. |
| RESULTS | | | |
| Study selection | 17 | Give numbers of studies screened, assessed for eligibility, and included in the review, with reasons for exclusions at each stage, ideally with a flow diagram. | Pages 13, 15, 17, and 20, first paragraph. Supplementary information tables from 1 to 5. |
| Study characteristics | 18 | For each study, present characteristics for which data were extracted (e.g., study size, PICOS, follow-up period) and provide the citations. | Pages 13, 15, 17, and 20, first paragraph. Supplementary information tables from 1 to 5. |
| Risk of bias within studies | 19 | Present data on risk of bias of each study and, if available, any outcome-level assessment (see Item 12). | Pages 10, 11, 12, 13, 14, 15, 17, 18, 20, 21. Tables from 1 to 4 in the article. Supplementary information tables from 1 to 5. |
| Results of individual studies | 20 | For all outcomes considered (benefits or harms), present, for each study: (a) simple summary data for each intervention group and (b) effect estimates and confidence intervals, ideally with a forest plot. | Supplementary information figures from 1 to 4. |
| Synthesis of results | 21 | Present results of each meta-analysis done, including confidence intervals and measures of consistency. | No meta-analysis was done. Page 6, fifth paragraph. |
| Risk of bias across studies | 22 | Present results of any assessment of risk of bias across studies (see Item 15). | Formal assessment not applicable because of the insufficient number of studies available. Page 6, fifth paragraph. |
| Additional analysis | 23 | Give results of additional analyses, if done (e.g., sensitivity or subgroup analyses, meta-regression [see Item 16]). | No additional analyses were performed. |
| DISCUSSION | | | |
| Summary of evidence | 24 | Summarize the main findings including the strength of evidence for each main outcome; consider their relevance to key groups (e.g., health care providers, users, and policy makers). | Pages 14, 16, 19, 22, 23, and 24. Supplementary information tables from 1 to 5. |
| Limitations | 25 | Discuss limitations at study and outcome level (e.g., risk of bias), and at review level (e.g., incomplete retrieval of identified research, reporting bias). | Limitations of the reviewed studies: Pages 13, 14, 16, 17, 18, and 20.  Tables from 1 to 4 in the article. Supplementary information tables from 1 to 5.  The limitations of our studies are reported at page 5, third paragraph. |
| Conclusions | 26 | Provide a general interpretation of the results in the context of other evidence, and implications for future research. | Pages 23 and 24. |
| FUNDING | | | |
| Funding | 27 | Describe sources of funding for the systematic review and other support (e.g., supply of data); role of funders for the systematic review. | Page 25. |
